# Cholinergic neuronal activity promotes diffuse midline glioma growth through muscarinic signaling

**DOI:** 10.1016/j.cell.2025.05.031

**Published:** 2025-06-19

**Authors:** Richard Drexler, Antonia Drinnenberg, Avishai Gavish, Belgin Yalçin, Kiarash Shamardani, Abigail E. Rogers, Rebecca Mancusi, Vrunda Trivedi, Kathryn R. Taylor, Yoon Seok Kim, Pamelyn J. Woo, Neeraj Soni, Minhui Su, Alexandre Ravel, Eva Tatlock, Alexandra Midler, Samuel H. Wu, Charu Ramakrishnan, Ritchie Chen, Alberto E. Ayala-Sarmiento, David Rincon Fernandez Pacheco, La’Akea Siverts, Tanya L. Daigle, Bosiljka Tasic, Hongkui Zeng, Joshua J. Breunig, Karl Deisseroth, Michelle Monje

**Affiliations:** 1 Department of Neurology and Neurological Sciences, Stanford University, Stanford, CA 94305, USA; 2 Department of Bioengineering, Stanford University, Stanford, CA 94305, USA; 3 Department of Psychiatry and Behavioral Sciences, Stanford University, Stanford, CA, USA; 4 Board of Governors Regenerative Medicine Institute, Cedars-Sinai Medical Center, Los Angeles, CA 90048, USA; 5 Allen Institute for Brain Science, Seattle, WA, USA; 6 Howard Hughes Medical Institute, Stanford, CA 94305, USA; 7 These authors contributed equally; 8 Lead contact

## Abstract

Glutamatergic neuronal activity promotes proliferation of both oligodendrocyte precursor cells (OPCs) and gliomas, including diffuse midline glioma (DMG). However, the role of neuromodulatory brainstem neurons projecting to midline structures where DMGs arise remains unexplored. Here, we demonstrate that midbrain cholinergic neuronal activity modulates OPC and DMG proliferation in a circuit-dependent manner. Optogenetic stimulation of the cholinergic pedunculopontine nucleus (PPN) promotes glioma growth in pons, while stimulation of the laterodorsal tegmentum nucleus (LDT) drives proliferation in thalamus. DMG-bearing mice exhibit higher acetylcholine release and increased cholinergic neuronal activity over the disease course. In co-culture, cholinergic neurons enhance DMG proliferation, and acetylcholine directly acts on DMG cells. Single-cell RNA sequencing revealed high CHRM1 and CHRM3 expression in primary DMG samples. Pharmacological or genetic blockade of M1/M3 receptors abolished cholinergic activity-driven DMG proliferation. Taken together, these findings demonstrate that midbrain cholinergic long-range projections promote activity-dependent DMG growth, mirroring a parallel proliferative effect on healthy OPCs.

## In brief

Midbrain cholinergic neuronal activity promotes the proliferation of both oligodendrocyte precursor cells and diffuse midline glioma (DMG) cells in a circuit-dependent manner, mediated by muscarinic M1 and M3 receptors in DMG; in turn, DMG increases midbrain cholinergic neuronal activity.

## Graphical abstract

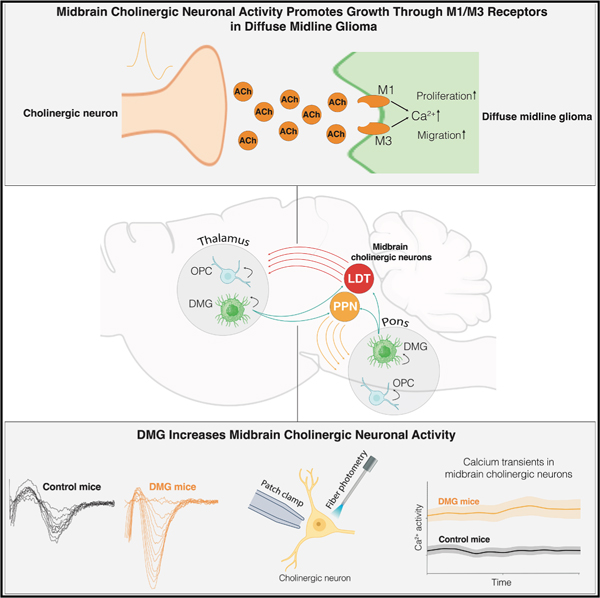

## INTRODUCTION

The activity of the nervous system has emerged as a crucial driver of cancers (for reviews, please see Monje et al.,^1^ Mancusi and Monje,^2^ and Winkler et al.^3^), and powerfully drives the initiation,^4,5^ and growth^6–10^ of primary brain cancers called gliomas. DMGs, the majority of which are driven by oncogenic mutations in genes encoding histone H3 (H3K27M), originate commonly in the pons and are also referred to as diffuse intrinsic pontine glioma (DIPG). H3K27M-altered DMGs also occur in the thalamus and spinal cord.^11^ DMG tumors originating in each location often spread throughout the midline and prominently involve the brainstem.^12–14^ Regardless of their specific location in these midline structures, DMGs arise from and closely resemble oligodendroglial lineage precursors,^11,15–20^ and numerous mechanistic parallels exist between the regulation of oligodendrocyte precursor cells (OPCs) and their malignant counterparts (for review, please see Taylor and Monje^21^). OPCs communicate extensively with neurons, both through neuron-to-OPC synapses^22,23^ and via paracrine factors such as BDNF.^24^ Neuronal activity promotes the proliferation of OPCs, oligodendrogenesis, and adaptive myelin changes that contribute to a range of functions including memory and learning in the healthy brain^24–29^ (for reviews on myelin plasticity please see Taylor and Monje^21^ and Knowles et al.^30^). Similarly, neuronal activity also drives glioma proliferation^6,8–10,31^ and invasion.^32,33^ The mechanisms mediating these growth-promoting effects of neurons on glioma cells include activity-regulated paracrine factor signaling^6,7,10^ and direct neuron-to-glioma synaptic communication.^8–10,31,32^ Glutamatergic, AMPA-receptor-mediated neuron-to-glioma synapses are found in both glioblastoma and DMGs,^8–10,32^ while depolarizing GABAergic neuron-to-glioma synapses are found more selectively in DMGs.^31^

To date, research efforts have chiefly focused on the influence of glutamatergic^4,6,8–10,32,33^ and GABAergic^31,34^ neurons on glioma pathophysiology, while the effects of neuromodulatory neuron types remain largely unexplored apart from the therapeutic potential of targeting dopamine receptor subtypes in glioblastoma.^35^ Neuromodulatory circuits are particularly salient to DMGs, given their frequent brainstem location and the robust axonal projections to midline structures of most neuromodulatory nuclei of the central nervous system. (The term ‘‘nucleus’’ in this context refers to clusters of neurons that work together toward a particular neurological function.) In particular, cholinergic midbrain nuclei such as the laterodorsal tegmentum nucleus (LDT) and pedunculopontine nucleus (PPN) project to midline structures where DMGs arise, highlighting the potential for these cholinergic neurons to modulate DMG pathophysiology. Here, we test this hypothesis and discover that cholinergic neuronal activity drives the proliferation of both healthy OPCs and DMG cells in a circuit-dependent manner.

## RESULTS

### Cholinergic neuronal activity increases OPC proliferation

**Figure 1.**
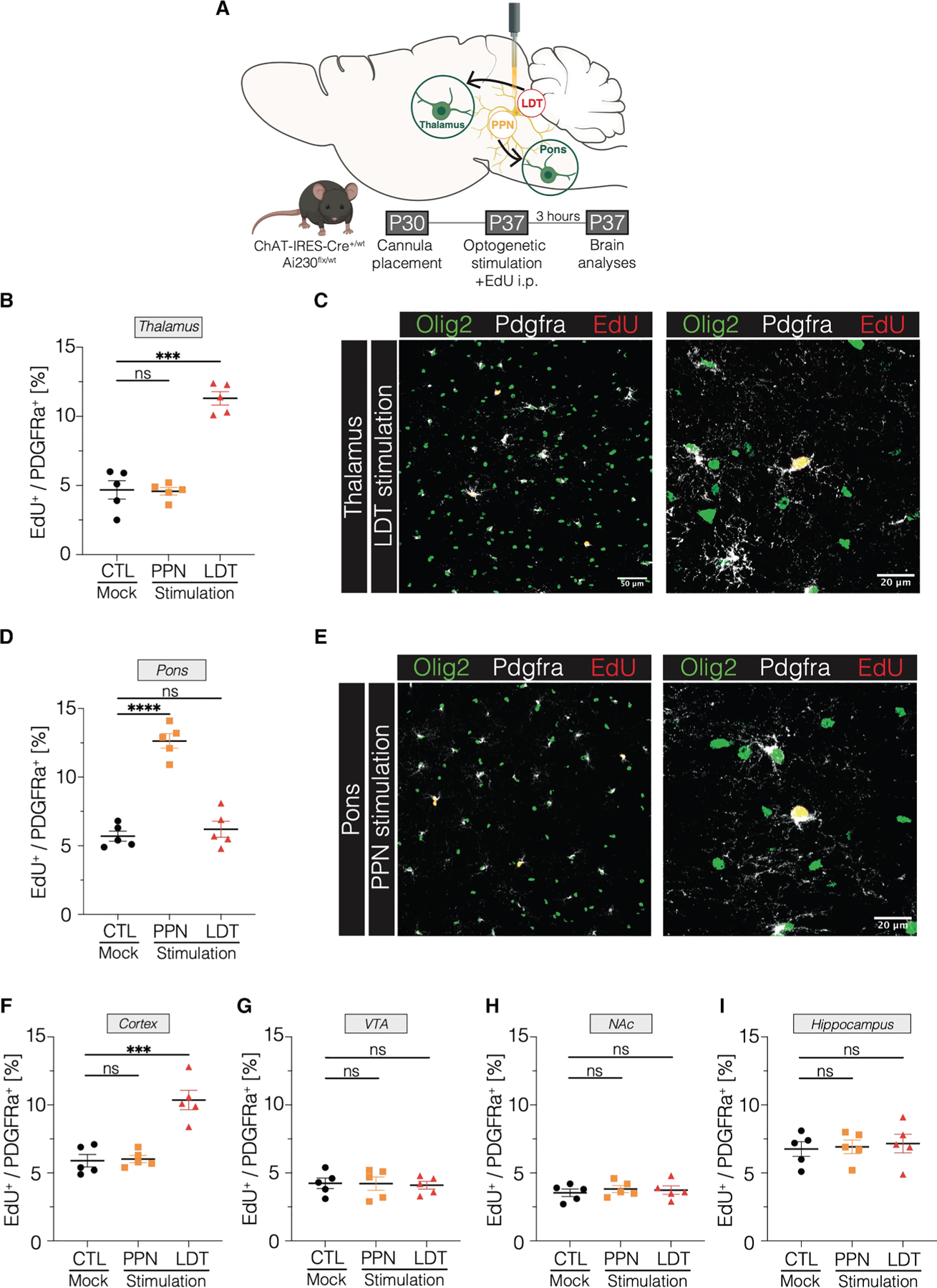
Cholinergic neuronal activity-regulated modulation of oligodendrocyte precursor cells (A) Schematic of experimental paradigm for optogenetic stimulation of cholinergic neurons in either laterodorsal tegmentum nucleus (LDT) or pedunculopontine nucleus (PPN). (B) Optogenetic stimulation of cholinergic neurons in LDT increases OPC proliferation (EdU^+^/Pdgfra^+^) in thalamus (CTL [not stimulated], PPN stimulated, and LDT stimulated, *n* = 5 mice/group). One-way analysis of variance (ANOVA) with Tukey’s post hoc analysis; ***p < 0.001, ns: non-significant. Data = mean ± SEM. (C) Confocal micrographs show thalamic OPC response after LDT stimulation. Olig2, green; Pdgfra, white; EdU, red, scale bars, 50 μm (left image) and 20 μm (right image). (D) Optogenetic stimulation of cholinergic neurons in PPN increases OPC proliferation (EdU^+^/Pdgfra^+^) in pons (CTL, PPN, and LDT, *n* = 5 mice). One-way ANOVA with Tukey’s post hoc analysis; ***p < 0.001, ns, non-significant. Data = mean ± SEM. (E) Confocal micrographs show pontine OPC response after PPN stimulation. Olig2, green; Pdgfra, white; EdU, red, scale bars, 50 μm (left image) and 20 μm (right image). (F–I) OPC response in (F) prefrontal cortex, (G) ventral tegmental area, (H) nucleus accumbens, and (I) hippocampus (CTL, PPN, and LDT, *n* = 5 mice/group). One-way ANOVA with Tukey’s post hoc analysis; **p < 0.01, ns, non-significant. Data = mean ± SEM. Related to Figures S1 and S2.

As neuronal activity influences both OPCs^24,26–28^ and DMGs,^6–8,10,31^ and given the resemblance of DMG cells to OPCs,^15,16,20^ we first explored the question of whether cholinergic neuronal activity modulates healthy OPC proliferation. We leveraged *in vivo* optogenetic stimulation to specifically target midbrain cholinergic neurons in either the PPN or the LDT (Figure 1A), which respectively project to the pons and thalamus. To stimulate cholinergic neurons, we generated a new TIGRE2.0^36^ transgenic reporter line, Ai230 (TIT2L-XCaMPG-ICL-ChRmine-TS-oScarlet-Kv2.1-IRES2-tTA2), that provides Cre-dependent expression of the potent channelrhodopsin ChRmine^37,38^ fused to the red fluorophore oScarlet, with the capacity for dual ChRmine-oScarlet/XCaMP-G^39^ reporting in the presence of Cre and tTA (STAR Methods, Figures S1A–S1C). ChRmine-oScarlet expression is enriched at the soma using the Kv2.1 motif.^40^ To specifically express ChRmine in cholinergic neurons, we crossed ChAT-IRES-Cre mice with Ai230 mice (ChAT-IRES-Cre^+/wt^ × Ai230^flx/wt^), thereby enabling stimulation of the potent opsin ChRmine^38,41^ specifically expressed in cholinergic neurons. We placed an optical ferrule within either the PPN or LDT (Figures S2A and S2B) to selectively stimulate cholinergic neurons confined to a single cholinergic nucleus. Light stimulation of the PPN or LDT at 20 Hz (595 nm, 30-min stimulation session with cycles of ten 15-ms pulses every 5 s) increased cFos expression, measured at 90 min, demonstrating successful optogenetic stimulation (Figures S2C–S2E). As cholinergic projections originating from the PPN and LDT target distinct brain regions, we performed optogenetic stimulation of each nucleus and evaluated OPC proliferation in response to cholinergic neuronal activity. Stimulation of cholinergic neurons increased OPC proliferation locally in the corresponding stimulated nuclei (Figures S2F and S2G), a pattern previously described for activity-regulated OPC proliferation.^26,42^ Furthermore, stimulation of the LDT increased OPC proliferation in the thalamus (Figures 1B and 1C), whereas PPN stimulation increased OPC proliferation in the pons (Figures 1D and 1E), as indicated by the quantification of EdU^+^/Pdgfra^+^ cells and consistent with the projection map of each midbrain cholinergic nucleus. These results illustrate circuit-dependent OPC proliferation consistent with target sites of midbrain cholinergic axonal projections.

Cholinergic axons from LDT and PPN also project to a variety of neuroanatomical sites. We found that LDT stimulation increased OPC proliferation in the cortex (Figure 1F), with no changes in OPC proliferation observed in the ventral tegmental area (VTA) (Figure 1G), nucleus accumbens (Figure 1H), or hippocampus (Figure 1I). This demonstrates a brain-region-specific response of OPCs to cholinergic neuronal activity, consistent with the heterogeneous and region-specific OPC responses previously demonstrated for dopaminergic neurons^42^ and glutamatergic cortical projection neurons.^26^

### Cholinergic neuronal activity stimulates glioma growth

Given the similarities between OPCs and DMG, and the observed circuit-dependent thalamic and pontine OPC responses to cholinergic neuronal activity, we next asked whether DMG cells are equally responsive to cholinergic neuronal activity in these midline structures. We combined Cre-dependent immunocompetent mouse models (ChAT-IRES-Cre) with an *in utero* electroporation-induced genetic mouse model of H3K27M-DMG that leverages dual recombinase-mediated cassette exchange to express mutations in *H3f3a*, *Tp53*, and *Pdgfra* in neural precursor cells (MADR).^43^ After generating tumor cells via *in utero* electroporation, DMG cells were cultured and subsequently allografted into immunocompetent mice. This model faithfully recapitulates H3.3-K27M DMG with OPC-like characteristics of the malignant cells previously described in the MADR model.^43^

To assess the general responsiveness of DMG cells to the activity of cholinergic axons, we expressed the excitatory opsin Channelrhodopsin (ChR2) specifically in cholinergic axons using an AAV viral vector strategy to target cholinergic axons (AAV injected in the thalamus or pons) and then stimulated cholinergic axons within the tumor microenvironment of pontine and thalamic allografts (Figures S3A and S3B). This strategy expressed ChR2 retrogradely in LDT and PPN cholinergic neurons, as well as the sparse cholinergic neurons in the pons and thalamus. In both locations, optogenetic stimulation of cholinergic axons resulted in an increased proliferation rate of DMG cells (Figures S3C and S3D), indicating that glioma growth is responsive to cholinergic axonal activity. Next, we expressed ChR2 in midbrain cholinergic projection axons anterogradely by injecting the viral vector in the PPN or LDT nuclei and then performed terminal field optogenetic stimulation of cholinergic axonal inputs to pons and thalamus (Figure S3E). This revealed a cholinergic nucleus-specific effect on growth; optogenetic stimulation of cholinergic axon projections from the PPN increased proliferation of pontine DMG cells (Figure S3F), while those from the LDT increased proliferation of thalamic DMG cells (Figure S3G).

To further elucidate the roles of each midbrain cholinergic nucleus and their effects on glioma cells, we proceeded with targeted stimulation of each cholinergic nucleus, using the Ai230 mouse model. DMG cells were allografted to either PPN or LDT nuclei in 4-week-old mice (ChAT-IRES-Cre^+/wt^ × Ai230^flx/wt^, post-natal day [P] 28–30), with optical ferrule placement in the respective nucleus 3 weeks later (P51). Optogenetic stimulation of cholinergic neurons was conducted after 4 weeks of tumor growth (P58). Mice were perfused 24 h after optogenetic stimulation (Figure S4A). Stimulation of cholinergic neurons in both midbrain nuclei increased the proliferation of DMG cells in the midbrain (Figures S4B–S4D), underscoring the glioma growth-promoting effects of cholinergic neuronal activity in the local tumor microenvironment.

### Cholinergic circuit-dependent effects on DMG

**Figure 2.**
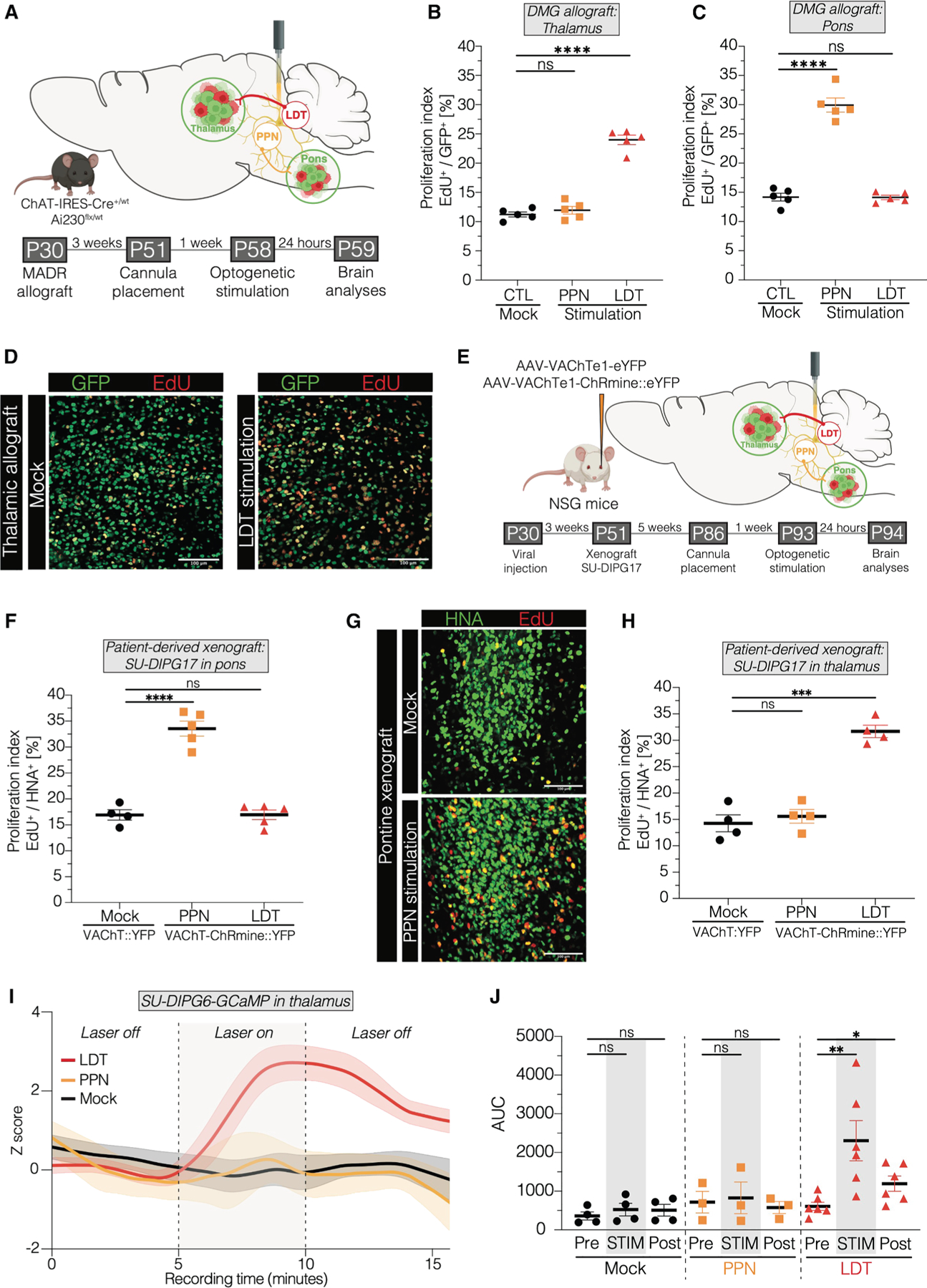
Cholinergic neuronal activity-mediated DMG proliferation (A) Schematic of experimental paradigm for optogenetic stimulation of cholinergic neurons in either LDT or PPN in mice bearing H3K27M DMG. (B) Proliferation index (EdU^+^/GFP^+^) of thalamic allografts in mice either stimulated in PPN or LDT or mock-stimulated (‘‘CTL’’) (CTL, PPN, and LDT, *n* = 5 mice/group). One-way analysis of variance (ANOVA) with Tukey’s post hoc analysis; ****p < 0.0001, ns, non-significant. Data = mean ± SEM. (C) Proliferation index (EdU^+^/GFP^+^) of pontine allografts in mice either stimulated in PPN or LDT or mock-stimulated (CTL) (CTL, PPN, and LDT, *n* = 5 mice/group). One-way ANOVA with Tukey’s post hoc analysis; ****p < 0.0001, ns, non-significant. Data = mean ± SEM. (D) Confocal micrographs showing proliferating GFP^+^ tumor cells in mock-stimulated (CTL) (upper images) and LDT-stimulated (‘‘LDT’’) (bottom images) mice. GFP, green; EdU, red, scale bars, 100 μm. (E) Schematic of experimental paradigm for xenografting with optogenetic stimulation of either LDT or PPN in immunodeficient mice. (F) Proliferation index (EdU^+^/HNA^+^) of pontine xenografts in mice either stimulated in PPN or LDT or mock-stimulated (‘‘Mock’’) (Mock, PPN, and LDT, *n* = 5 mice/group). One-way ANOVA with Tukey’s post hoc analysis; ****p < 0.0001, ns: non-significant. Data = mean ± SEM. (G) Confocal micrographs illustrate proliferating HNA^+^ glioma cells in mock-stimulated (Mock) (upper images) and PPN-stimulated (‘‘PPN’’) (bottom images) mice after xenografting into pons. HNA, green; EdU, red; scale bars, 100 μm. (H) Proliferation index (EdU^+^/HNA^+^) of thalamic xenografts in mice either stimulated in PPN or LDT or mock-stimulated (Mock) (Mock, PPN, *n* = 4 mice/group; and LDT, *n* = 5 mice/group). One-way ANOVA with Tukey’s post hoc analysis; ***p < 0.001, ns: non-significant. Data = mean ± SEM. (I) Fiber photometry recordings showing averaged GCaMP-labeled calcium transients in thalamic glioma cells with simultaneous mock (*n* = 4 mice), LDT (*n* = 6 mice), and PPN (*n* = 3 mice) stimulation. (J) AUC of fiber photometry recordings from (I) before, during, and after stimulation of the cholinergic nuclei. One-way ANOVA with Tukey’s post hoc analysis; **p < 0.01, *p < 0.05, ns: non-significant. Data = mean ± SEM. Related to Figures S3 and S4.

Next, we allografted DMG cells into the thalamus or pons, followed by stimulation of either the midbrain cholinergic LDT or PPN nucleus (Figure 2A). As observed for OPCs, stimulation of cholinergic neurons in the LDT increased cell proliferation in thalamic allografts more than 2-fold (Figures 2B–2D); no discernible effect was observed in the pons after LDT stimulation (Figure 2C). Concordantly, stimulation of PPN cholinergic neurons increased DMG cell proliferation in pontine allografts, but not in thalamic allografts (Figures 2B and 2C). This circuit-dependent effect on DMG growth dynamics was further supported by using an inhibitory optogenetic approach, expressing halorhodopsin (eNpHR 3.0) via viral vector to inhibit neuronal activity in LDT and PPN by hyperpolarization of cholinergic neurons. Optogenetic inhibition of LDT or PPN midbrain cholinergic neurons led to a circuit-specific decrease in thalamic and pontine glioma cell proliferation, respectively (Figures S4E–S4G).

Patient-derived orthotopic xenograft models of DMG are complimentary to genetically engineered mouse models. We next tested the effects of cholinergic neuronal activity on patient-derived H3.3-K27M DMG orthotopically xenografted to the pons or thalamus. To express the red-shifted opsin ChRmine in cholinergic neurons in an immunodeficient mouse model amenable to xenografting, viral vectors (AAV.PHP.eB-VAChTe1-ChRmine:: eYFP or AAV.PHP.eB-VAChTe1-eYFP) were retro-orbitally injected into 4-week-old NOD-SCID-IL2-gamma chain-deficient (NSG) mice (Figures S4H and S4I), followed by xenografting of patient-derived DMG cells (SU-DIPG17). Evaluation of cell-type specificity for cholinergic neurons in the PPN and LDT under these conditions yielded specificity of 72.2% (Figure S4I and STAR Methods). Optogenetic stimulation of midbrain cholinergic nuclei was performed after 6 weeks of tumor engraftment, with perfusion conducted 24 h later (Figure 2E). Consistent with the results above, stimulation of the PPN increased the proliferation of patient-derived DMG cells xenografted into the pons, whereas this effect in pons was not observed following LDT stimulation (Figures 2F and 2G). Conversely, LDT stimulation increased proliferation in thalamic DMG cells (Figure 2H).

To monitor real-time calcium signaling dynamics in patient-derived DMG cells—a known driver of glioma progression^10,44^—in response to cholinergic neuronal activity, we xenografted DMG cells expressing a GCaMP6s sensor (SU-DIPG6-GCaMP6s) into the thalamus of NSG mice that had been previously injected retro-orbitally with a viral vector to express ChRmine or control construct in cholinergic neurons (Figures S4J and S4K). Fiber photometry recordings enabling measurements of *in vivo* calcium transients revealed an increase in glioma cell calcium transients in response to cholinergic neuronal activity, dependent on the specific nucleus being stimulated (Figures 2I and 2J), further highlighting this circuit-dependent interaction.

Taken together, these findings mirror the OPC proliferative response described above and highlight midbrain cholinergic neuronal activity as a driver of DMG proliferation throughout the cholinergic circuitry, including within the midbrain and in projections to the thalamus and pons.

### Neurotrophin signaling in cholinergic neuronal-activity-dependent DMG growth

**Figure 3.**
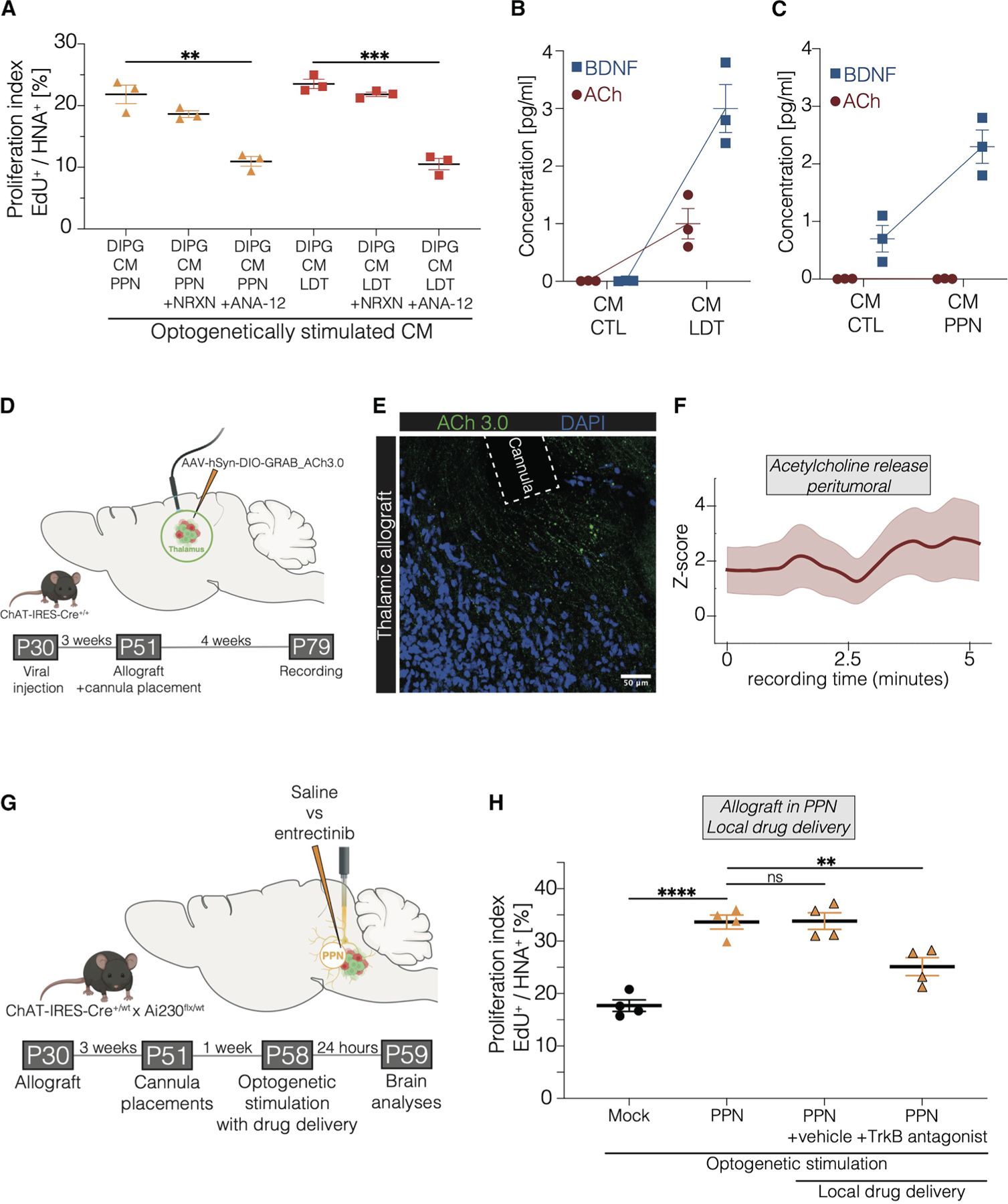
Mechanisms of activity-regulated midbrain cholinergic neuron-to-glioma signaling (A) Quantification of glioma cell proliferation (EdU^+^/HNA^+^) in DMG cells exposed to conditioned medium (CM) from LDT or PPN midbrain explants following cholinergic neuronal optogenetic stimulation. One-way analysis of variance (ANOVA) with Tukey’s post hoc analysis; **p < 0.01, ***p < 0.001. Data = mean ± SEM; *n* = three independent experiments, each with three wells per condition; each data point represents the mean of three wells per condition for a given experiment. (B and C) Measurement of BDNF and acetylcholine levels in midbrain explant CM from (B) LDT and (C) PPN. Data = mean ± SEM; *n* = three independent experiments, each with three wells per condition; each data point represents the mean of three wells per condition for a given experiment. (D and E) Schematic of the experimental paradigm and representative image showing the recording site for GRAB-ACh3.0 sensor experiment, performed to measure acetylcholine release in the peritumoral microenvironment of thalamic allografts. ACh3.0, green; DAPI, blue; scale bar, 50 μm. (F) Average GRAB-ACh3.0 fluorescence at the 4-week time point following thalamic allograft implantation (*n* = 6 mice). (G) Schematic of the experimental paradigm used to assess the local effects of BDNF-TrkB signaling in activity-regulated cholinergic neuron-to-glioma signaling in the midbrain. (H) Proliferation index (EdU^+^/HNA^+^) of DMGallografts in the PPN in mice with PPN optogenetic stimulation and treated with either vehicle or entrectinib delivered locally to the tumor (*n* = 4 mice/group). One-way ANOVA with Tukey’s post hoc analysis; ****p < 0.0001, **p < 0.01, ns: non-significant. Data = mean ± SEM. Related to Figures S5 and S6.

Given previous studies demonstrating various activity-regulated paracrine signals that promote glioma cell proliferation,^4–6,10^ we investigated the role of secreted factors in cholinergic neuronal activity-regulated DMG proliferation. Conditioned medium (CM) was collected from midbrain explants containing either PPN or LDT nuclei (Figure S5A). *Ex vivo* optogenetic stimulation of cholinergic neuronal cell bodies in either PPN or LDT explants generated CM that increased patient-derived DMG proliferation *in vitro* (Figures S5B and S5C). The CM was fractionated by molecular size, which revealed that the proliferative effect of the midbrain explant CM is attributable to macromolecules with a molecular weight between 10 and 100 kDa (Figure S5D), consistent with previous studies that identified neuroligin-3 (NLGN3)^4,6,7^ and BDNF^4,6,10^ as paracrine factors promoting glutamatergic neuronal activity-regulated glioma proliferation in cortex^6^ and optic nerve.^4^ ANA-12 (a specific inhibitor of the BDNF-receptor TrkB) abolished the proliferative effect of cholinergic neuronal activity-regulated factors in PPN-CM or LDT-CM while the addition of Neurexin (to sequester NLGN3) only minimally decreased proliferation (Figure 3A). Concordant with the conclusion that BDNF is the chief paracrine growth factor released as a result of midbrain cholinergic neuronal activity in this experimental paradigm, elevated BDNF levels were found in LDT-CM (Figure 3B) and PPN-CM (Figure 3C), while NLGN3 was only mildly increased in midbrain cholinergic nuclei CM by western blot analysis (Figure S5E). Acetylcholine levels were mostly unchanged in midbrain explant CM following cholinergic neuronal activity (Figures 3B and 3C). These midbrain cholinergic nuclei explants contain cholinergic cell bodies but not cholinergic projection axon terminals, which likely explains the lack of acetylcholine release into CM in this experimental paradigm.

To investigate acetylcholine release from thalamic axon terminals of cholinergic neurons *in vivo*, we conducted fiber photometry recordings using a GRAB-ACh3.0 sensor (Figures 3D and 3E). This allowed us to measure acetylcholine release from cholinergic axon terminals within the DMG microenvironment of the thalamus (Figure 3F). Acetylcholine release, together with activity-regulated neurotrophin signaling from midbrain cholinergic neurons to TrkB receptors on DMG cells, may both contribute to cholinergic neuronal activity-driven tumor growth, although the relative contribution of each may vary depending on tumor location.

To test the role of BDNF-TrkB signaling in cholinergic activity-regulated DMG proliferation in the midbrain, we allografted DMG cells in the PPN and administered a TrkB antagonist (Entrectinib) directly to the tumor 30 min prior to optogenetic stimulation using an optofluid cannula for intratumoral drug delivery (Figure 3G). Local delivery of the TrkB antagonist decreased glioma cell proliferation in the midbrain (Figure 3H), though not to baseline levels, highlighting both the contribution of BDNF-TrkB signaling in cholinergic neuronal activity-dependent DMG growth in the midbrain and the existence of multiple activity-regulated mechanisms.

To examine whether this effect is confined to the midbrain where the cholinergic neuron somato-dendritic compartment is located or extends to cholinergic neuron axonal projections, we xenografted a patient-derived DMG model with high migratory potential (SU-DIPG13FL)^13^ into the midbrain, allowing it to spread to multiple anatomical regions, including the pons, thalamus, PPN, and LDT, over 10 weeks (Figures S6A–S6C). Mice received a TrkB antagonist systemically for 7 consecutive days before perfusion to evaluate anti-proliferative effects in specific brain compartments. No optogenetic stimulation was performed in this experiment. TrkB-antagonist-treated mice exhibited significantly reduced malignant cell proliferation in the pons, thalamus, and cholinergic midbrain nuclei (Figures S6D–S6H), demonstrating that BDNF-TrkB signaling affects DMG cells broadly and equivalently throughout midline neuroanatomical compartments, consistent with the complex neuronal environment of these midline structures and the previous findings that BDNF released as a result of the activity of other neuron types (e.g., glutamatergic neurons) robustly promotes DMG growth.^6,10^

### Acetylcholine directly affects DMG growth and migration

**Figure 4.**
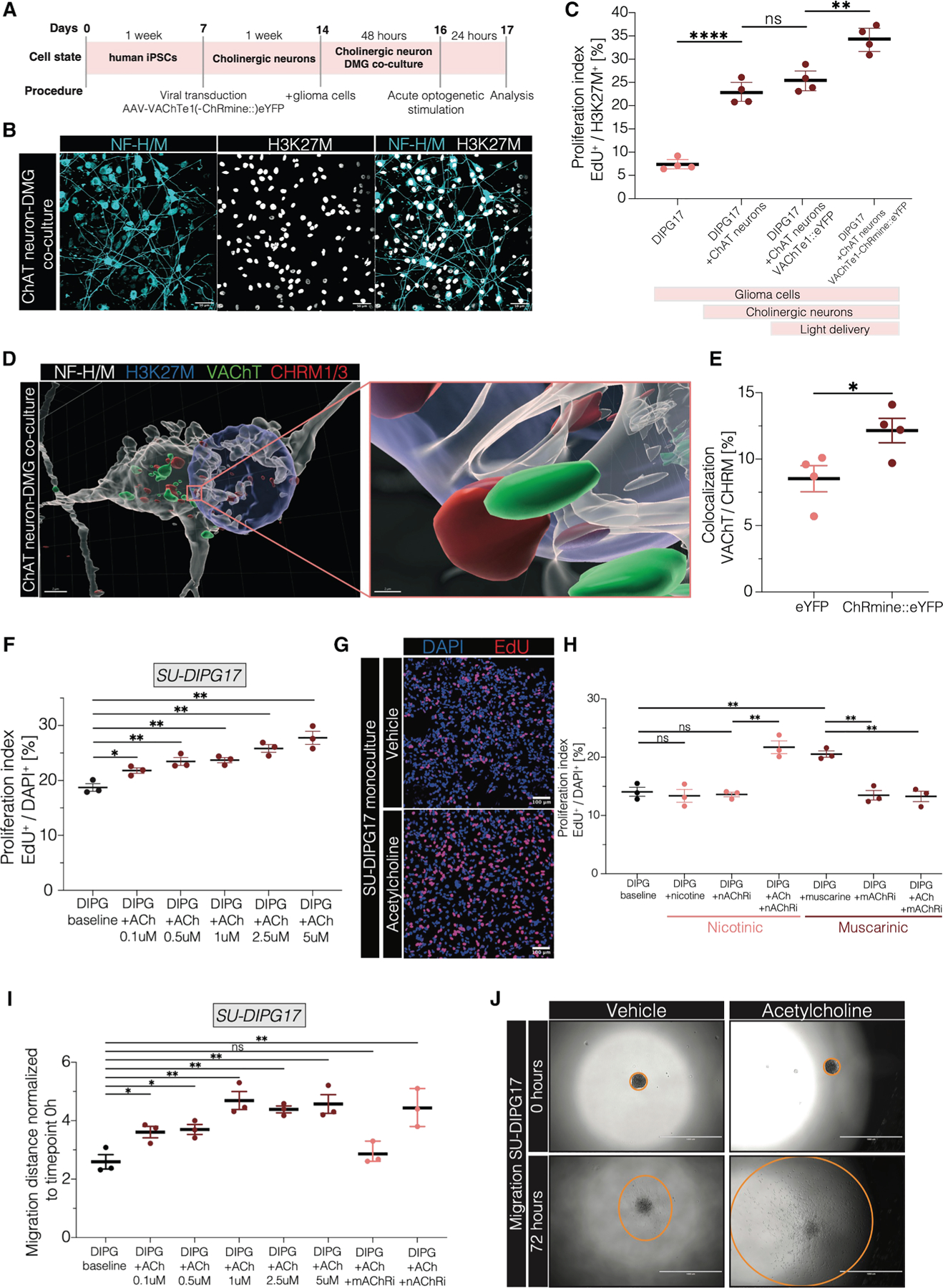
Direct effects of acetylcholine on DMG (A) Timeline for the generation of iPSC-derived cholinergic with viral transduction (AAV-VAChTe1-ChRmine::eYFP or AAV-VAChTe1::eYFP) for co-culturing with glioma cells. (B) Confocal micrographs showing cholinergic motor neurons generated hiPSCs of a healthy 12-year-old male and co-cultured with DMG cells of an 8-year old male. NF-H/M, turquoise; H3K27M, white; scale bars, 50 μm. (C) Quantification of glioma cell proliferation (EdU^+^/H3K27M^+^) when co-cultured with hiPSC-derived cholinergic motor neurons paired with *in vitro* optogenetic stimulation. One-way analysis of variance (ANOVA) with Tukey’s post hoc analysis; ****p < 0.0001, **p < 0.01, ns: non-significant. Data = mean ± SEM; *n* = four independent experiments, each with three wells per condition; each data point represents the mean of three wells per condition for a given experiment. (D) Three-dimensional rendering illustrating presynaptic cholinergic neuron (NF-H/M: white) with presynaptic puncta (VAChT: green) co-localizing with postsynaptic puncta (CHRM1/3: red) expressed by post-synaptic glioma cell (H3K27M: blue) in a cholinergic neuron-DMG co-culture. Scale bars, 2 μm. (E) Co-localization of VAChT (presynaptic cholinergic neurons, indicated by NF-H/M^+^) and CHRM1/3 (post-synaptic glioma cells, indicated by H3K27M^+^) in the DMG cell line when co-cultured with hiPSC-derived cholinergic motor neurons. Unpaired two-tailed Welch’s t test; *p < 0.05. Data = mean ± SEM; *n* = four independent experiments, each with three wells per condition; each data point represents the mean of three wells per condition for a given experiment. (F) Proliferation index (EdU^+^/DAPI^+^) of a patient-derived DMG cell line (SU-DIPG17) after exposure to different concentrations of acetylcholine. One-way ANOVA with Tukey’s post hoc analysis; **p < 0.01, *p < 0.05, ns: non-significant. Data = mean ± SEM; *n* = three independent experiments, each with three wells per condition; each data point represents the mean of three wells per condition for a given experiment. (G) Representative confocal micrographs showing the proliferation of a patient-derived cell line after exposure to vehicle control (top image) or 5 μM acetylcholine (bottom image). DAPI, blue; EdU, red; scale bars, 100 μm. (H) Proliferation index (EdU^+^/DAPI^+^) of a patient-derived cell line (SU-DIPG17) after exposure to various muscarinic and nicotinic receptor agonists (nicotine and muscarine) and antagonists (mecamylamine and scopolamine). One-way ANOVA with Tukey’s post hoc analysis; **p < 0.01, ns: non-significant. Data = mean ± SEM; *n* = three independent experiments, each with three wells per condition; each data point represents the mean of three wells per condition for a given experiment. (I) 3D migration assay analysis. One-way ANOVA with Tukey’s post hoc analysis; *p < 0.05, **p < 0.01. Data = mean ± SEM; *n* = three independent experiments, each with three wells per condition; each data point represents the mean of three wells per condition for a given experiment. (J) Representative images showing the glioma cell migration at timepoint zero and after 72 h. Scale bars, 1,000 μm. Related to Figures S7 and S8.

We next co-cultured patient-derived DMG cells with cholinergic neurons derived from human induced pluripotent stem cells (hiPSCs). hiPSC-derived cholinergic motor neurons from a healthy 12-year-old male were matched with a pontine DMG cell culture from an 8-year-old male (SU-DIPG17) (Figures 4A and 4B). We transfected hiPSC-derived cholinergic motor neurons with a viral vector (AAV.PHP.eB-VAChTe1-ChRmine:: eYFP or AAV.PHP.eB-VAChTe1-eYFP) to enable optogenetic stimulation *in vitro*. Co-culture with DMG cells resulted in a 2-fold increase in the DMG cell proliferation rate, with an even greater proliferation rate observed following optogenetic activation of cholinergic neurons (Figure 4C). Cholinergic neuron-to-DMG synaptic structures were evident both *in vitro* (Figure 4D) and *in vivo* (Figures S7A and S7B). The number of synaptic structures increased after optogenetic stimulation of cholinergic neurons *in vitro* (Figure 4E).

To assess the direct effects of acetylcholine, we tested the proliferation rate of DMG cell cultures (*n* = 4 patient-derived cultures, Table S1) exposed to varying concentrations of acetylcholine. These experiments revealed a dose-dependent increase in DMG cell proliferation (Figures 4F and 4G; Figure S7C–S7E). Muscarinic receptor blockers abrogated the effects of acetylcholine, while nicotinic receptor blockers had no effect on the proliferation caused by acetylcholine (Figure 4H). Neither muscarinic nor nicotinic receptor blockers affected DMG cell proliferation in the absence of acetylcholine (Figure 4H). Furthermore, muscarine (a direct muscarinic receptor agonist) induced cell proliferation to the same extent as acetylcholine, while nicotine (a direct nicotinic receptor agonist) exerted no effect (Figure 4H). Taken together, these data indicate that the proliferative effect of acetylcholine is mediated by muscarinic receptors rather than nicotinic receptors.

Given the neuromodulatory functions of acetylcholine,^45^ we next asked whether acetylcholine alters the known^8,10^ proliferative effect of cortical neuron co-culture on glioma cells. Early post-natal mouse pup (P0-P1) cortical preparation generated mixed glutamatergic (Vglut^+^) and GABAergic (GAD65^+^) cortical neuron cultures (Figure S7F). As expected,^8,10^ cortical neuron-DMG co-culture markedly increased the proliferation rate of DMG cells (Figures S7G–S7I). Addition of acetylcholine substantially augmented this effect, further increasing the glioma cell proliferation rate in a dose-dependent manner (Figures S7H and S7I).

Midbrain cholinergic neurons are known to co-release other neurotransmitters, including glutamate and GABA, to some extent. Both neurotransmitter signaling pathways have been shown to induce glioma cell membrane depolarization, leading to subsequent proliferation.^10,31^ To investigate whether these additional neurotransmitters contribute to cholinergic neuronal activity-induced DMG cell proliferation, we inhibited glutamatergic signaling via blocking AMPA receptors and GABAergic signaling via blocking GABA_A_ receptors *in vitro* (Figures S8A–S8C), and pharmacologically inhibiting AMPAR signaling *in vivo* via local drug delivery (Figures S8D and S8E). The proliferative effects of cholinergic neuronal activity on DMG cells were unaffected by pharmacological inhibition of glutamatergic or GABAergic signaling (Figures S8C–S8E), indicating that the impact of cholinergic activity on DMG proliferation is independent of these pathways.

Previous studies indicate that neuronal activity promotes the widespread dissemination of glioma cells.^32,33^ Concordantly, we found that acetylcholine exposure increased the migration of DMG cells *in vitro* in a neurotransmitter dose-dependent manner (Figures 4I and 4J). Taken together, these findings highlight a direct effect of acetylcholine on proliferation and infiltration in DMG.

### CHRM1 and CHRM3 mediate effects of cholinergic neuronal activity in DMG

**Figure 5.**
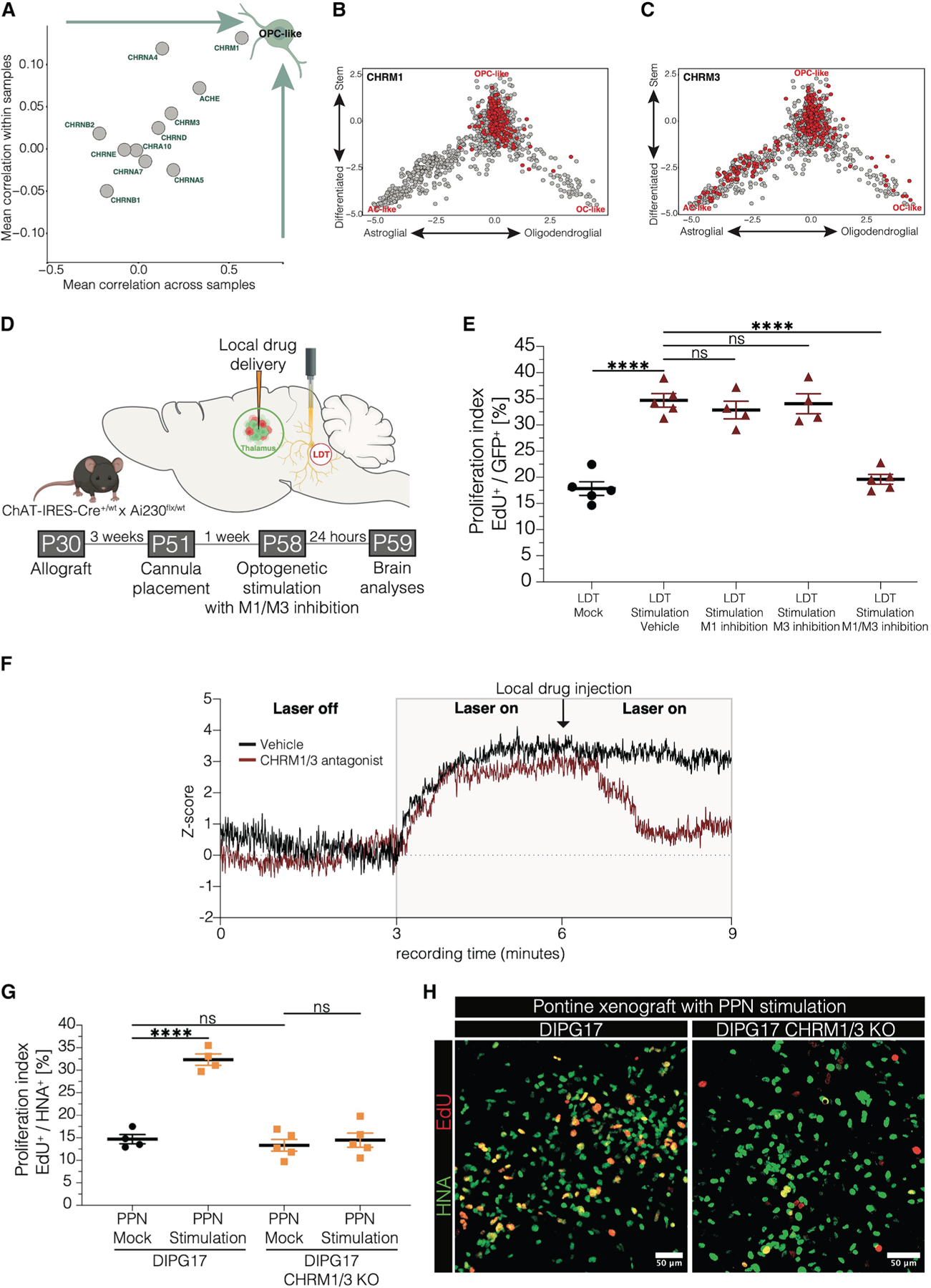
CHRM1 and CHRM3 mediate the effects of cholinergic neuronal activity in pontine and thalamic DMG (A) Scatter plot correlating cholinergic receptor gene expression values with an OPC-like score in DMG samples. (B) Two-dimensional representation of the association between *CHRM1* expression (red dots: centered value > 1) and the OPC-like (y axis) as well as OC-like and AC-like (x axis) scores for H3K27M DMGs. (C) Two-dimensional representation of the association between *CHRM3* expression (red dots: centered value > 1) and the OPC-like (y axis) as well as OC-like and AC-like (x axis) scores for H3K27M DMGs. (D) Schematic of the experimental paradigm for local antagonism of muscarinic receptors M1 and M3 in thalamic allografts. (E) Proliferation index (EdU^+^/GFP^+^) of thalamic allografts in mice optogenetically stimulated in LDT or mock-stimulated controls, with or without administration of M1 (VU0255035) or M3 receptors (4-DAMP) pharmacological inhibitors. ‘‘Mock,’’ ‘‘Vehicle,’’ and ‘‘M1/M3 inhibition’’ *n* = 5 mice/group; ‘‘M1 inhibition’’ and ‘‘M3 inhibition’’ *n* = 4 mice/group. One-way analysis of variance (ANOVA) with Tukey’s post hoc analysis; ****p < 0.0001, ns: non-significant. Data = mean ± SEM. (F) Fiber photometry recordings showing averaged GCaMP-labeled calcium transients in thalamic glioma cells with simultaneous LDT stimulation and either local vehicle or CHRM1/3 antagonist infusion (*n* = 4 mice/group). (G) Proliferation index (EdU^+^/HNA^+^) of pontine xenografts in mice optogenetically stimulated in the PPN or mock-stimulated controls, with either control (Cas9-expressing) patient-derived DMG cells (DIPG17) or dual CHRM1/3 CRISPR-mediated knockout (DIPG17-CHRM1/3-KO). ‘‘DIPG17’’ *n* = 4 mice/group; ‘‘DIPG17-CHRM1/3-KO’’ *n* = 5 mice/group. One-way ANOVA with Tukey’s post hoc analysis; ****p < 0.0001, ns: not significant. Data = mean ± SEM. (H) Confocal micrographs showing proliferating HNA^+^ tumor cells in the pons of either control (Cas9-expressing) patient-derived DMG cells (DIPG17) or dual CHRM1/3 CRISPR-mediated knockout cells (DIPG17-CHRM1/3-KO) in PPN-stimulated mice. HNA, green; EdU, red, scale bars, 50 μm. Related to Figures S10.

The proliferation-promoting effects of acetylcholine *in vitro* and cholinergic long-range projections *in vivo* raise questions about the cholinergic receptor profile of DMG cells. We analyzed receptor gene expression in single-cell and single-nucleus RNA sequencing (sc/snRNA-seq) datasets from primary patient tumor samples across various central nervous system tumors, including 54 DMG samples. Cholinergic receptor genes exhibited varying levels of expression across malignant cells within DMG tumors (Figure S9A), reflecting intratumoral heterogeneity—a phenomenon well-described in this disease^11^ and in gliomas in general.^46–48^ Pseudo-bulk expression in malignant cells revealed that the muscarinic receptor CHRM3 was highly expressed across all glioma subtypes, as well as in ependymoma and medulloblastoma (Figure S9B). Analysis of differentially expressed genes associated with either muscarinic or nicotinic receptor genes showed a specific association between muscarinic receptor genes and *PDGFRA*—a hallmark gene for OPCs—exclusively in DMG (Table S2). We further tested correlations between cholinergic receptor gene expression and OPC scores, both within tumor cells and across samples at the pseudo-bulk level. We found that the muscarinic receptor gene *CHRM1* exhibited the highest values on both measures, stronger than the values for the muscarinic receptor gene *CHRM3* (Figure 5A). Both *CHRM1* and *CHRM3* expression were enriched in the OPC-like cell state of DMG (Figures 5B and 5C). Further comparisons underscored the association between *CHRM1* and OPC-like states in DMG using Neftel et al.^46^ and a pan-cancer study-derived cell states^49^ as references (Figure S9C). While not the most highly expressed cholinergic receptor gene in glioma, *CHRM1* seems to play a unique role in OPC-like DMG cells, distinguishing it from other CNS tumor entities such as glioblastoma, ependymoma, and medulloblastoma.

Since CHRM1 and CHRM3 appear to play significant roles in DMG, we investigated their involvement in DMG proliferation both *in vitro* and *in vivo*. In a co-culture model, we found that delivery of both M1 receptor (VU0255035) and M3 receptor (4-DAMP) antagonists was necessary to abolish the proliferation-inducing effects of cholinergic neurons on DMG (Figures S10A and S10B). The reduction in the DMG proliferation rate by M1/M3 antagonists was further observed following *in vitro* optogenetic stimulation of cholinergic neurons (Figure S10C). Blocking M1/M3 also led to a decrease in the number of synaptic structures between cholinergic neurons and DMG cells (Figure S10D).

*In vivo* experiments confirmed that blocking both receptors was required to reduce DMG cell proliferation in the thalamus following LDT stimulation (Figures 5D and 5E). Fiber photometry recordings of GCaMP-transduced DMG cells, combined with local drug delivery to the glioma cells, revealed a robust decrease in calcium signaling during ongoing optogenetic LDT stimulation with M1/M3 blockade (Figure 5F). Since muscarinic receptors can be present at both pre- and post-synaptic sites, which may be blocked by pharmacological inhibition, we performed a CRISPR-based knockout of both muscarinic receptors in a patient-derived DMG xenograft model to specifically block the muscarinic receptors M1 and M3 in the glioma cells. This experiment validated the findings above, as the effect of cholinergic neuronal activity on glioma cell proliferation in the pons was completely abrogated in the M1/M3 knockout DMG cells following PPN cholinergic neuronal stimulation *in vivo* (Figures 5G and 5H).

Taken together, these findings highlight the critical and redundant role of muscarinic receptors M1 and M3 in activity-dependent cholinergic neuron-DMG interactions.

### DMG cells increase midbrain cholinergic neuronal activity

**Figure 6.**
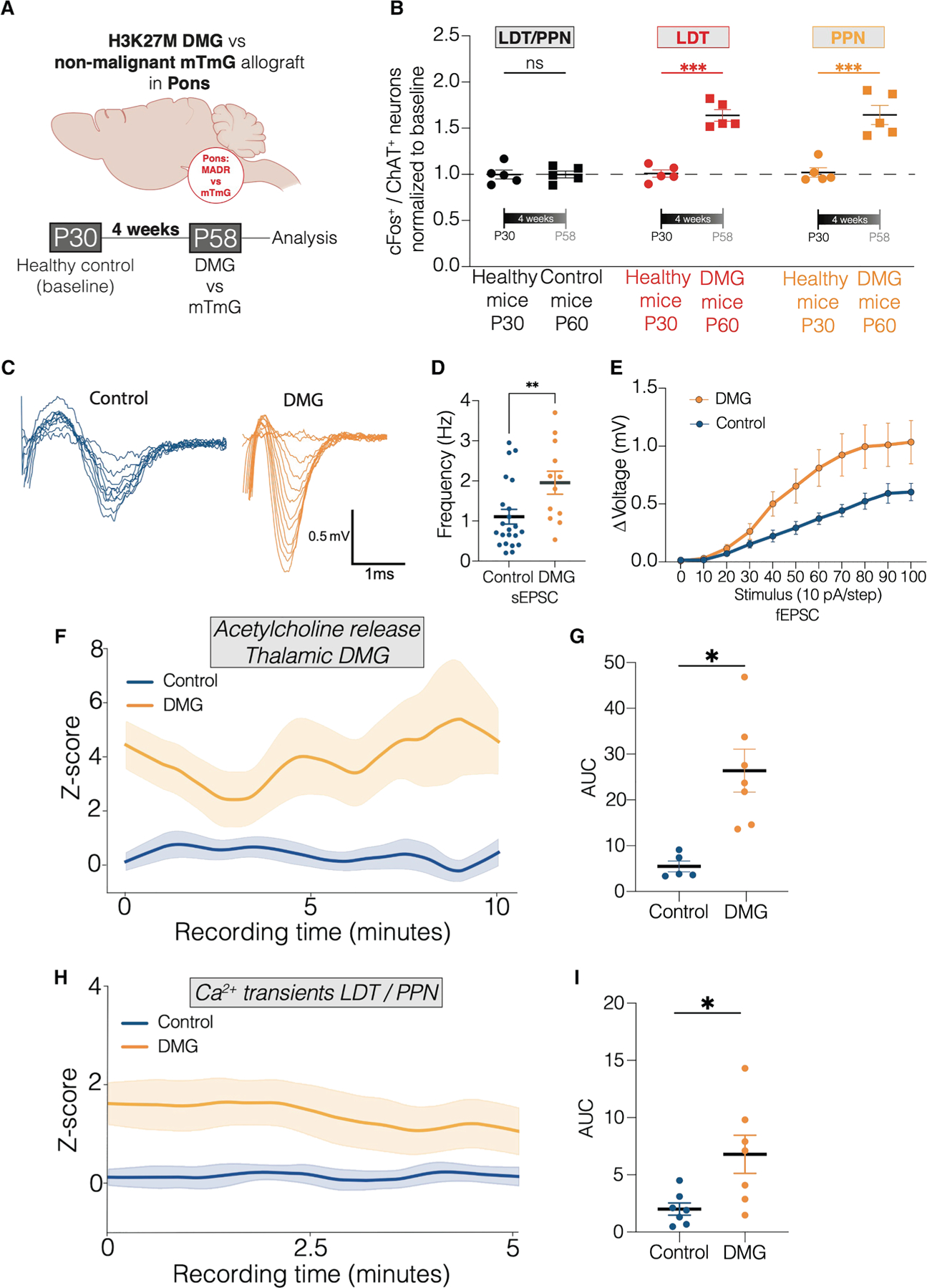
Diffuse midline glioma increases midbrain cholinergic neuronal activity (A) Schematic of experimental paradigm for investigating reciprocal signaling effects of tumor cells to midbrain cholinergic neurons. (B) Neuronal activity (cFos^+^/ChAT^+^ neurons) in LDT and PPN of pontine tumor-bearing mice when compared with control-injected mice (*n* = 5 mice/group). Unpaired two-tailed Welch’s t test; ***p < 0.001, ns: non-significant. Data = mean ± SEM. (C) The sample trace shows the output field potential comparison between the control and DMG-bearing mice. (D and E) sEPSCs (D) were recorded from cholinergic neurons within the LDT. Population data show a significant increase in sEPSC frequency in MADR-bearing mice compared with control-injected mice (MADR: 2.0 ± 0.29 Hz, *n* = 12, 5 mice; Control 1.1 ± 0.18 Hz, *n* = 22, 6 mice). Data shown as mean, error bars indicate SEM; *p* = 0.0083 (α = 0.05). Statistical analysis was performed using a two-tailed nonparametric t test with Mann-Whitney correction. fEPSC (E) data were compared between control and DMG-bearing mice, revealing a significant increase in output field potentials in MADR mice compared to controls (two-way ANOVA mixed-effect analysis, *p* <0.0001, α = 0.05). (F) Average GRAB-ACh3.0 fluorescence in the peritumoral environment at the 2-week time point following thalamic implantation of DMG (orange line, *n* = 7 mice) or control cells (blue line, *n* = 5 mice). (G) AUC of GRAB-ACh3.0 recordings from (F) of DMG or control-injected animals. Unpaired two-tailed Welch’s t test; *p < 0.05. Data = mean ± SEM. (H) Fiber photometry recordings showing averaged GCaMP-labeled calcium transients in cholinergic neurons of LDT and PPN from DMG (*n* = 8 mice) or control-injected (*n* = 7 mice) animals. Measured fluorescence is combined from both the LDT and PPN. (I) AUC of fiber photometry recordings from (H) in DMG or control-injected animals. Measured fluorescence is combined from both the LDT and PPN. Unpaired two-tailed Welch’s t test; *p < 0.05. Data = mean ± SEM. Related to Figure S11.

Glioma cells secrete paracrine factors that increase neuronal hyperexcitability in the cortex and hippocampus^8,50,51^ and cause remodeling of functional neural circuits.^52^ This led us to hypothesize that midbrain cholinergic neurons may be reciprocally influenced by DMGs. We injected either DMG cells (‘‘MADR’’) or control cells (Neuro-2a mouse neuroblast cells expressing the mTmG construct but no oncogenes,^43^ ‘‘mTmG’’) into the pons or thalamus of 4-week-old immunocompetent mice (ChAT-IRES-Cre^+/wt^ × Ai230^flx/wt^) and assessed cholinergic neuronal activity by expression of the immediate early gene cFos within both midbrain cholinergic nuclei after 4 weeks of tumor growth (Figure 6A; Figure S11A). We observed increased midbrain cholinergic neuronal activity within both nuclei in pontine tumor-bearing mice, which was not observed in vehicle control-injected mice (Figure 6B). This finding was further validated in thalamic allografts (Figure S11B).

Electrophysiological recordings of cholinergic neurons within the LDT (Figure S11C) at the same time point revealed elevated field potentials (Figure 6C), along with significantly increased spontaneous (sEPSCs) (Figure 6D) and frequency-evoked excitatory post-synaptic currents (fEPSCs) (Figure 6E) in tumor-bearing mice, indicating heightened midbrain cholinergic neuronal activity in the presence of a tumor distant from the neuronal somas. How tumor cells in one region influence the excitability of neurons whose somas are located some distance from the cancer remains to be fully understood, but this could involve retrograde effects transmitted through axonal projections or by secreted factors acting at a distance.

We further employed a GRAB sensor to monitor acetylcholine levels over time (Figure S11D). A significant increase in acetylcholine release was detected 2 weeks post-tumor implantation within the tumor microenvironment of DMG-bearing mice, compared with controls (Figures 6F and 6G), with release levels continuing to rise with time (Figures S11E and S11F). This highlights the temporal dynamics of increasingly elevated acetylcholine release in midline structures during tumor progression.

We next conducted fiber photometry using the genetically encoded calcium sensor GCaMP6m to measure real-time calcium signaling in the PPN and LDT in freely moving mice (Figure S11G). Cholinergic neuronal soma calcium transients were significantly elevated in the PPN and LDT 2 weeks following DMG allograft (Figures 6H and 6I), with further increases in cholinergic neuronal calcium transients over time as the tumor progressed (Figures S11H and S11I).

Taken together, these findings indicate bidirectional interactions between pontine or thalamic DMG and midbrain cholinergic neurons, where cholinergic neuronal activity promotes DMG growth, and DMG cells increase cholinergic neuronal activity in a temporally dynamic manner.

## DISCUSSION

H3K27M-altered DMGs are aggressive central nervous system (CNS) cancers that occur in midline structures, chiefly the pons (also called DIPG), thalamus, and spinal cord. Both DMGs and OPCs—the DMG cell of origin^11,15–20^—proliferate in response to the activity of glutamatergic neurons.^6,26^ Here, we have tested the effects on healthy OPCs and malignant DMG cells of cholinergic neurons located in the midbrain. The axon projections of cholinergic neurons in the two distinct cholinergic nuclei of the midbrain—LDT and PPN—project robustly to the thalamus and pons, respectively. We found that both healthy OPCs and DMG cells in pons and thalamus proliferate in response to cholinergic neuronal activity in a circuit-dependent manner, with acetylcholine acting on muscarinic M1 and M3 acetylcholine receptors enriched in the OPC-like DMG malignant cellular subpopulation. Conversely, DMG cells induce hyperexcitability in cholinergic neurons, progressively increasing midbrain cholinergic neuronal activity over the disease course.

Given the similarities between healthy OPC and DMG cells,^11,15–19^ it is useful to study both in parallel (for review, see Taylor and Monje^21^). Similar to glioma cells,^6^ glutamatergic neuronal activity promotes the proliferation of healthy OPCs,^26^ as well as oligodendrogenesis and adaptive, activity-regulated myelin changes that tune neural circuit function.^26,29,53^ Here, we found that cholinergic long-range projections similarly influence both healthy and malignant cell proliferation. OPC proliferation can reflect either the generation of new oligodendrocytes, or a blockade in differentiation that maintains the precursor in a proliferating state. Cholinergic signaling in OPCs is complex: in cultured OPCs, muscarinic signaling promotes OPC proliferation,^54,55^ increased survival,^56^ and prevents differentiation,^57^ while nicotinic signaling promotes OPC differentiation into mature oligodendrocytes.^58^ Thus, acetylcholine acts through muscarinic signaling to block oligodendrogenesis while acting through nicotinic signaling to promote oligodendrogenesis. Concordantly, muscarinic blockade promotes remyelination^57,59^ after demyelinating injury, and muscarinic antagonists have been^60^ and continue to be tested in clinical trials for multiple sclerosis (NCT02521311 and NCT05338450). We find that cholinergic neuronal activity promotes OPC proliferation in midline brain regions and in cortex, but not in other regions such as the hippocampus and VTA reflecting the regional heterogeneity of OPCs.^61^

The proliferative effects of long-range cholinergic neuron projections to thalamus and pons are chiefly mediated through acetylcholine signaling via M1 and M3 receptors, as the DMG growth-promoting effects of cholinergic neuronal activity are fully blocked by a combination of M1 and M3 blockade *in vitro* and *in vivo*. This redundancy in M1 and M3 receptor function highlights the relative importance of cholinergic signaling to DMG cancer cells. Furthermore, cholinergic neuronal activity influences DMG cells in the midbrain through BDNF-TrkB signaling, which is known to promote proliferation and malignant synaptic plasticity in gliomas.^6,10^ Explants of cholinergic midbrain nuclei containing cholinergic neuronal soma and dendrites exhibited activity-regulated BDNF release that promoted DMG proliferation. These results do not rule out a possible contribution of BDNF release at cholinergic axon terminals in pons and thalamus, but as M1/M3 blockade completely abrogated the cholinergic neuronal activity-regulated increase in DMG proliferation at these sites—sites of acetylcholine release by midbrain cholinergic neurons—cholinergic neuron contributions to DMG growth in pons and thalamus appear to be dominated by acetylcholine signaling. Thus, while BDNF-TrkB signaling is certainly an important therapeutic target in DMG,^10^ the genetic and pharmacological muscarinic receptor blockade experiments here demonstrate that the chief mechanism of cholinergic neuronal activity-driven DMG proliferation in the pons and thalamus is mediated by acetylcholine through M1 and M3 receptors.

In line with recent evidence that cholinergic neurons can form synapses with glioblastoma cells,^62,63^ we found evidence of cholinergic neuron-to-DMG cell synaptic structures. These findings underscore the critical role of cholinergic neurons and their activity in DMGs, alongside the established roles of glutamatergic^8,10^ and GABAergic^31^ synaptic signaling. Neuron-to-DMG networks and the integration of DMG cells into neuronal circuitry thus includes cholinergic neurons.

Interactions between neurons and glioma are bidirectional, and glioma cells can profoundly affect neuronal function by increasing neuronal excitability^50,51,64–67^ and functionally remodeling neural circuits.^52^ We observed that DMG influences the activity of midbrain cholinergic neurons, which intensifies as glioma progression advances. Given the important neuromodulatory effects of cholinergic signaling on cognition and other brain functions (for review, see Picciotto et al.^45^), this raises the possibility that tumor-induced dysregulation of cholinergic neurons in the brainstem may contribute to the cognitive, behavioral, and emotional symptoms that DMG patients frequently experience.^68^ Further investigation into the interactions between neuromodulatory brainstem neurons and DMG tumor cells is essential, not only to elucidate these critical pathways but also to identify potential therapeutic strategies aimed at mitigating cognitive and psychiatric symptoms in patients with DMG.

Evaluation of primary tumor single-cell/single-nucleus datasets revealed the muscarinic receptor CHRM3 as the most highly expressed cholinergic receptor gene across various CNS tumors, including DMG. CHRM1 appears as a unique target that is upregulated in the OPC-like malignant cell state within DMG. Our findings highlight the potential of these two receptors as therapeutic targets for DMG. The nervous system regulates a wide range of cancers (for reviews, please see Mancusi and Monje,^2^ Winkler et al.,^3^ and Hanahan and Monje^69^), and emerging principles in the burgeoning field of Cancer Neuroscience are becoming evident. Cholinergic signaling through muscarinic receptors is required for glandular organogenesis^70^ and has emerged as a frequent mechanism regulating tumor pathophysiology, evident not only in brain tumors as explored here, but also in diverse cancer types including gastric,^71^ colon,^72^ and prostate cancers.^73^ Blocking M1 and M3 muscarinic receptors demonstrates the therapeutic potential of targeted muscarinic receptor inhibition for DMG. Beyond DMG, the literature indicates broad potential applications for clinical development of specific M1 and/or M3 inhibitors to target muscarinic signaling in cancer.

### Limitations of the study

Several areas remain open for future study: (1) we focused exclusively on cholinergic neurons of the midbrain but did not investigate cholinergic neurons of the basal forebrain. (2) Electrophysiological and electron microscopy studies should be performed to demonstrate bona fide cholinergic neuron-to-DMG synapses. Electrophysiology may also help determine whether additional receptor subunits beyond CHRM1 and CHRM3 contribute to putative cholinergic glioma synapses. (3) Our studies identified cholinergic neuronal-activity-mediated mechanisms of acetylcholine and BDNF release, but an unbiased screening approach may uncover additional cholinergic activity-regulated paracrine factors.

### Conclusions

Taken together, the results presented here implicate midbrain cholinergic neurons as an important driver of DMG pathophysiology. Each neuron type studied to date has proven to contribute to DMG growth and progression through targetable mechanisms. A comprehensive understanding of the neuroscience of DMG will enable the development of effective combination therapy strategies for these lethal central nervous system cancers of childhood.

## RESOURCE AVAILABILITY

### Lead contact

Further information and requests should be directed to the lead contact, Michelle Monje (mmonje@stanford.edu).

### Materials availability

The transgenic Ai230 (B6;129S6-Igs7<tm230(tetO-XCaMPG,CAG-ChRmine*/oScarlet*,-tTA2)Daigl>/J) mouse line is available through Jackson Laboratory (JAX #37944). Additionally, the viral vector AAV.PHP.eb eHGT_78h-Cre-PEST is available via Addgene (Addgene #231791). The newly generated AAV.PHP. eB-VAChTe1-ChRmine::eYFP viral vector is available upon request.

### Data and code availability

All analyzed single-cell and single-nucleus RNA sequencing data have been deposited by the study groups that generated the datasets. Raw, unprocessed confocal images used for 3D rendering in Main Figure 4D Figures S7A and S7B have been deposited at Mendeley at https://doi.org/10.17632/79bb48wfkw.1. Any additional information required to reanalyze the data reported in this paper is available from the lead contact upon request.

## STAR★METHODS

### EXPERIMENTAL MODEL AND STUDY PARTICIPANT DETAILS

Diffuse midline glioma (DMG) cultures were established from patient-derived surgical specimens obtained with informed consent, in accordance with protocols approved by the Stanford University Institutional Review Board (IRB). Single-cell RNA sequencing data from human DMG samples were obtained from previously published, fully anonymized datasets.

This study used different mouse strains. Both female and male mice were used equally across experiments. Mouse lines included ChAT-IRES-Cre, Ai230, and NOD-SCID IL2Rγ-deficient (NSG) strains. All procedures involving animals were conducted under protocols approved by the Stanford University Institutional Animal Care and Use Committee (IACUC) and in accordance with institutional and national guidelines. Mice were housed under standard conditions with a 12 h light/dark cycle, 21 °C ambient temperature, 60% humidity, and ad libitum access to food and water.

### METHOD DETAILS

#### Patient-Derived Diffuse Midline Glioma Cells

The utilized patient-derived glioma models included SU-DIPG-13fl, SU-DIPG-17, SU-DIPG-19, and SU-DIPG-92. Patient characteristics for these models are detailed in Table S1. Throughout the culture period, all cultures were subjected to monitoring for authenticity via short tandem repeat (STR) fingerprinting and routine mycoplasma testing was conducted. The glioma cultures were cultivated as neurospheres in serum-free medium composed of DMEM (Invitrogen), Neurobasal(-A) (Invitrogen), B27(-A) (Invitrogen), heparin (2 ng ml—1), human-bFGF (20 ng ml—1) (Shenandoah Biotech), human-bEGF (20 ng ml—1) (Shenandoah Biotech), human-PDGF-AA (10 ng ml—1) (Shenandoah Biotech), and human-PDGF-BB (10 ng ml—1) (Shenandoah Biotech). For *in vitro* experiments, the neurospheres were dissociated using TrypLE (Gibco) for seeding.

#### Generation of H3.3 K27M MADR cells

The H3.3 K27M MADR tumor cell cultures were generated using the same technique as previously described.^43,74^ Briefly, Gt(ROSA) 26Sortm4(ACTB-tdTomato,-EGFP)Luo/J and Gt(ROSA)26Sortm1.1(CAG-EGFP)Fsh/Mmjax mice (JAX Mice) were crossed with wild-type CD1 mice (Charles River) to produce heterozygous mice. Male and female embryos between E12.5 and E15.5 were subjected to *in utero* electroporation. Pregnant dams were individually housed, and pups remained with their mothers until P21 in the institutional animal facility (Stanford University). The MADR tumor cell line used here was generated by dissociating and sorting GFP^+^ tumor cells from female heterozygous mTmG mice. Subsequently, MADR cultures were maintained as neurospheres in serum-free medium composed of DMEM (Invitrogen), Neurobasal(-A) (Invitrogen), B27(-A) (Invitrogen), heparin (2 ng ml—1), human bFGF (20 ng ml—1) (Shenandoah Biotech), human bEGF (20 ng ml—1) (Shenandoah Biotech), human PDGF-AA (10 ng ml—1) (Shenandoah Biotech), insulin (Sigma-Aldrich), and 2-mercaptoethanol (Sigma-Aldrich). The non-malignant counterpart cells, mTmG cells, constitutively express tdTomato and were generated as described previously. mTmG cells (mouse neuroblasts engineered to express the mTmG construct without oncogenes) were cultured in medium composed of DMEM (Invitrogen), 10% fetal bovine serum (R&D Systems), GlutaMAX (Invitrogen), and Antibiotic-Antimycotic (Invitrogen).

#### Animal Models

Homozygous ChAT-IRES-Cre mice (created by Dr. Bradford Lowell and obtained from The Jackson Laboratory, strain 006410) were either used as a pure line with the genotype ChAT-IRES-Cre^+/+^ or bred with homozygous transgenic Ai230 mice (specified below). Optogenetic experiments using allograft diffuse midline glioma models were performed on animals with the genotype ChAT-IRES-Cre^+/wt^ x Ai230^flx/wt^, which enabled optogenetic control selectively for cholinergic neurons in immunocompetent mice. All mice used were genotyped at postnatal day 10. For allograft tumor studies, glioma cell implantation was conducted using an electroporated, engineered H3.3K27-altered MADR^43^ model. To replicate results obtained from this immunocompetent tumor model in patient-derived glioma cells, viral vectors (specified below) were injected into 4-week-old NSG mice (The Jackson Laboratory), allowing optogenetic control of the laterodorsal tegmentum nucleus and pedunculopontine nucleus in immunodeficient mice, followed by xenografting of patient-derived DIPG cells (SU-DIPG17 or SU-DIPG6-GCaMP6s) after 3 weeks of virus expression. For tumor implantation studies, allograft and xenograft experiments were performed under strict adherence to IACUC-defined morbidity criteria, which prioritize signs of neurological decline over tumor volume. Mice were immediately euthanized if they exhibited neurological symptoms or experienced a weight loss of ≥15% from their baseline.

#### Generation of Transgenic Ai230 Mice

##### Transgenic Reporter Mouse

Ai230 (B6;129S6-Igs7<tm230(tetO-XCaMPG,CAG-ChRmine*/oScarlet*,-tTA2)Daigl>/J, mice available on JAX #037944, plasmid TIT2L-XCaMPG-ICL-ChRmine-TS-oScarlet-Kv2.1-IRES2-tTA2 available on Addgene #234865) is a new TIGRE2.0 transgenic reporter^36^ designed for Cre-dependent expression of soma-targeted ChRmine-oScarlet^37,38^ and XCaMP-G.^39^ However, characterization of expression showed that XCaMP-G levels are minimal after Cre delivery only (Figure S1B). Additional hSynapsin promoter-driven viral tTA expression resulted in XCaMP-G expression (Figure S1C). Therefore, Ai230 can be used either as a Cre-dependent ChRmine-oScarlet reporter or as a Cre/tTA- dependent reporter for both ChRmine-oScarlet and XCaMP-G. Ai230 mice contain a modified TIGRE genomic locus that contains (5’ to 3’): FRT3 – 2X HS4 chicken beta globin insulators – Tet responsive 2 promoter comprised of seven repeats of TRE binding sites and a minimal CMV promoter (based on that in Clontech’s pTRE2-hyg vector) – *LoxP* – a stop cassette (with stops in all three frames linked to a synthetic pA-hGH pA-PGK pA unit) – *LoxP* – XCaMP-G –WPRE – bGH polyA – 2X HS4 chicken beta globin insulators – CAG promoter (which consists of the CMV enhancer fused to the chicken beta-actin promoter – Lox2272 – stop cassette (with stops in all three frames linked to a synthetic pA-hGH pA-TK pA unit) – Lox2272 – ChRmine-membrane trafficking signal (TS)-oScarlet-Kv2.1 – WPRE – bGH pA – IRES2 – tetracycline-transactivator 2 (tTA2) – WPRE – bGH polyA – PGK promoter – one domain of the hygromycin resistance gene – mRNA splice donor sequence – Frt5. The locus was generated by Flp recombinase-mediated cassette exchange into a previously made docking site integrated into the TIGRE locus^36^ in mouse embryonic stem (ES) cells. Correctly targeted ES cell clones were injected into fertilized blastocytes to obtain high percentage chimeras. Chimeras were then bred to C57BL/6J mice to achieve germline transmission. The resulting heterozygous TIGRE reporter mice were first maintained in a C57BL/6J congenic background, then crossed with each other to generate homozygous mice. To target ChRmine-oScarlet expression to cholinergic neurons, homozygous Ai230 mice were crossed with homozygous ChAT-IRES-Cre mice (JAX #006410).

#### Characterization of Ai230 Reporter Mouse

##### Viral Constructs

For AAV.PHP.eb-eHGT_78h-Cre-PEST (maps, sequence, and plasmid available at Addgene #231791), the excitatory enhancer eHGT_78h^75^ and the gene for Cre were cloned including a PEST sequence in an AAV2 backbone. For AAV2/5 hsyn-Cre-IRES-tTA, the construct hsyn-Cre-IRES-tTA was generated using standard cloning techniques. Both constructs were tested for expression in cultured neurons and packaged by the Stanford Neuroscience Gene Vector and Virus Core (GVVC). Prepackaged AAV PHP.eb eHGT_78h-Cre-PEST virus can be purchased from the Stanford GVVC.

##### Surgery and Intracranial Injections

To reduce inflammation, dexamethasone (2 mg/kg) was administered subcutaneously 2–3 hours prior to surgery. Heterozygous Ai230 mice (8–12 weeks) were anesthetized with a mixture of Ketamine (100 mg/kg) and Xylazine (10 mg/kg) and 0.5–1% isoflurane. The skull was exposed and a 5mm craniotomy was performed above visual cortex with the dura fully intact. A glass pipette (20 μm opening, beveled tip) was filled with AAV2/5 hsyn-Cre-IRES-tTA 3×10^11^ vg/ml. 500nl viral solution was injected at 1 nl/s using a Nanoinject III injector (Drummond Scientific, Catalog No. 13-681-460) ~350 μm below the brain surface in the primary visual cortex. The pipette was left in place for 5–10 minutes after injection and then slowly retracted. The craniotomy was closed with a 5mm glass coverslip, and a metal head bar was mounted using dental cement (Metabond). Buprenorphine sustained release (SR) was injected subcutaneously 30 minutes before the end of surgery.

##### Retro-Orbital Injections

A syringe (BD Micro Fine insulin syringe 0.5 ml, 30G) was front-loaded with AAV PHP.eb eHGT_78h-Cre-PEST (2×10^11^ viral particles per mouse, 40 μl total volume injected per mouse). Heterozygous Ai230 mice aged 4–5 weeks were anesthetized with isoflurane and injected into the retro-orbital sinus from the nasal side. Histology was performed 4 weeks after the injection.

##### Two-photon Imaging

Awake, head-fixed imaging was conducted using a commercial two-photon microscope (Ultima 2P plus). A 16X0.8 NA objective (Nikon) was immersed in diluted ultrasound gel for imaging. For XCaMP-G and oScarlet imaging, 500 frames were acquired using 920nm and 980nm excitation, respectively. Imaging data was registered using suite2p before the mean fluorescence image was computed.

##### Histology, Immunohistochemistry and Confocal Imaging

Mice were anaesthetized with isoflurane and Beuthanasia-D (100 mg/kg i.p.) and transcardially perfused with 20 ml of ice-cold PBS followed by 50 ml of 4% PFA in PBS. Brains were extracted and post-fixed in 4% PFA for 24 hours at 4° C before transferring to 30% sucrose in PBS for cryoprotection overnight at 4 °C. Coronal sections (40 μm) were cut on a freezing microtome and stored in cryoprotectant at 4° C. The cryoprotectant solution consisted of 250 ml glycerol, 300 ml ethylene glycol, and 450 ml 1X PBS (pH adjusted to 6.7 with 1 M HCl). Sections were washed three times in PBS for 10 minutes each and then incubated for 1hr in blocking buffer (PBS +0.3% TritonX and 5% normal donkey serum) at room temperature. Sections were incubated with rabbit anti-GFP (Thermo Fisher Scientific A11122) diluted 1:500 in blocking buffer overnight at 4°C. Sections were washed three times in PBST for 30 minutes each and incubated with the secondary antibody donkey anti-rabbit IgG conjugated with Alexa-647 (Thermo Fisher Scientific A31573) and DAPI (1:1000 dilution) for two hours at room temperature, diluted 1:500 in blocking buffer, followed by three washes of 30 minutes each in PBST. Confocal imaging was performed using a Zeiss confocal scanning laser microscope (LSM 880) with a 20X objective.

#### VAChTe1 Enhancer-based Promoter Sequence Design

We used the CATlas genome browser^76^ to identify genomic regions with enhancer activity predominantly in cholinergic neurons, specifically in CHAT-VIP and CHAT-Pallidal-Striatal subtypes and exhibiting high sequence conservation with the human genome near the cholinergic marker gene SLC18A3 (VAChT). Based on this analysis, we identified a conserved candidate enhancer in mice, which we termed VAChTe1 (coordinates: chr14:32,464,311–32,465,371, mm10). The corresponding human sequence (chr7:155,592,223–155,605,565, hg19) was synthesized for this study.

#### Viral VAChTe1 Constructs

The plasmids pAAV-VAChTe1-ChRmine-eYFP-WPRE and pAAV-VAChTe1-eYFP-WPRE were constructed by replacing the CaMKIIa promoter with the VAChTe1 promoter sequence into pAAV-CaMKIIa-ChRmine-eYFP-WPRE^37^ or pAAV-CaMKIIa-eYFP-WPRE respectively, using MluI and BamHI restriction sites. The plasmids were fully sequence-verified through whole plasmid sequencing. AAV-PHP.eB viruses were produced by the Stanford Neuroscience Gene Vector and Virus Core.

#### Allografting and Xenografting

Male and female mice were used in cohorts equally. For optogenetic stimulation studies, MADR cultures (*H3.3 K27 MADR line 1, 200,000 cells per mice*) or patient-derived DMG cultures (*SU-DIPG17*, *SU-DIPG13fl,* and *SU-DIPG6-GCaMP6s,* 400,000 cells per mouse) were injected into the thalamus or pontine region. For investigating the reciprocal effects from thalamic or pontine DMG cells to midbrain cholinergic neurons, control mTmG cells (*200,000 cells per mouse*) were injected as controls to MADR cells. A single-cell suspension of all cultures was prepared in sterile culture medium immediately before surgery. Animals at P28–P35 were anaesthetized with 1–4% isoflurane and placed on stereotactic apparatus. Under sterile conditions, the cranium was exposed via a midline incision and a 31-gauge burr hole made at exact coordinates. For thalamus injections the coordinates were as follows: AP=−1.0mm (from bregma), ML=+0.8mm, DV=−3.5mm. For pontine injections coordinates were AP=−0.8 (from lambda), ML=−1.0mm, DV=−5.0mm. For local injections into the LDT and PPN, the coordinates mentioned below were used (see ‘*Stereotaxic Surgery, Ferrule Placement, and Viral Vectors*’). Cells were injected using a 31-gauge Hamilton syringe at an infusion rate of 0.5 μl min—1 with a digital pump. At completion of infusion, the syringe needle was allowed to remain in place for a minimum of 5 minutes, then manually withdrawn. The wound was closed using 3M Vetbond (Thermo Fisher Scientific) and treated with Neo-Predef with Tetracaine Powder.

#### Stereotaxic Surgery, Ferrule Placement, and Viral Injection

Mice were anesthetized with 1–4% isoflurane and placed in a stereotaxic apparatus. For optogenetic experiments targeting midbrain cholinergic neurons, mice were unilaterally implanted with optical fibers positioned in the laterodorsal tegmentum nucleus (LDT) or the pedunculopontine nucleus (PPN) on the right side. These optical fibers were positioned into the LDT at the following coordinates (measured from bregma): AP = −5.0 mm, ML = +0.5 mm, DV = −3.15 mm. For PPN stimulation or inhibition, the coordinates were AP = −4.70 mm, ML = +1.25 mm, DV = −3.50 mm. Optical fibers for stimulation of thalamic and pontine local cholinergic neurons, and for fiber photometry recordings, were placed unilaterally at the coordinates outlined below. All optical fibers were secured with stainless steel screws (thread size 00–90 × 1/16, Antrin Miniature Specialties) placed contralateral to the fiber, along with C&B Metabond and light-cured dental adhesive cement (Geristore A&B paste, DenMat). Viral vectors were unilaterally injected into the region of interest using a Hamilton Neurosyringe and Stoelting stereotaxic injector over a period of 5 minutes.

#### Retro-Orbital Injections of AAV.PHP.eB-VAChTe1-ChRmine::eYFP

To replicate findings using patient-derived DMG cultures in immunodeficient NSG mice, we performed retro-orbital injections of newly constructed AAV.PHP.eB-VAChTe1-ChRmine::eYFP or AAV.PHP.eB-VAChTe1-eYFP. For intravascular targeting of midbrain cholinergic neurons, 4-week-old mice were injected retro-orbitally with 60 μL of virus solution (3 × 10^12^ vg/ml in 60 μL 0.9% NaCl per mouse). Mice were anesthetized with isoflurane and placed in a prone position on a heating pad to maintain body temperature. The injection site, located at the medial canthus of the eye, was cleaned with 70% ethanol. Using a 30-gauge needle, the virus solution was carefully injected into the retro-orbital sinus. The specificity of AAV.PHP.eB-VAChTe1-ChRmine::eYFP for cholinergic neurons was confirmed using confocal microscopy with immunostaining for ChAT and analysis of ChAT + YFP co-localization in LDT and PPN. Co-localization of YFP signal with ChAT-positive and ChAT-negative neurons in the LDT and PPN demonstrated a specificity of 72.2% across 107 neurons from 5 mice. As with many enhancers, validation is required for each preparation, as specificity is sensitive to transduction condition; we noted that acceptable specificity was observed with retro-orbital systemic delivery of the construct as used here and assessed by immunohistochemistry (shown in Figure 4H, 60 μL of 3 × 10^12^ vg/ml PHPeb virus, followed by spatially localizing the transduced cells) but not when the construct was delivered intracranially at high concentrations (500 nL of 5 × 10^11^ vg/ml PHPeb virus and assessed by hairpin chain reaction).

#### Optogenetic Stimulation

Optogenetic stimulations were performed one week after optic ferrule implantation. For experiments involving optogenetic stimulation of cholinergic neurons in the LDT and PPN regions of ChAT-IRES-Cre^+/wt^ x Ai230^flx/wt^ mice or NSG mice using the previously described AAV-VAChTe1-ChRmine::eYFP construct, freely moving animals were connected to a 595 nm high-power LED system via a monofiber patch cord to achieve stimulation of ChRmine. For experiments with local or terminal field stimulation of cholinergic projections in the glioma microenvironment, a 473 nm diode-pumped solid-state laser system was employed to stimulate ChR2. Cholinergic neuron stimulation, for both neuronal cell bodies and axon terminals, was performed at 20 Hz, ten 15 ms pulses of 595 nm light delivery every 5 seconds at a light power output of 10mW from the tip of the optic fiber (200 μm core diameter, NA=0.22 - Doric lenses). Optogenetic stimulation sessions lasted for 30 minutes. A 20 Hz stimulation frequency was previously shown to effectively increase cholinergic neuron firing rates and proved effective for optogenetic activation of cholinergic neurons.^77–80^ Using a 10 mW output resulted in a power density of 9.72 mW/mm^2^ across the cholinergic nuclei, based on an approximate nucleus size of 0.8 mm,^81^ ensuring full activation of each nucleus. Power density calculations were performed according to the methodology described by Yizhar et al.^82^

Animals were injected intraperitoneally with 40 mg/kg EdU (5-ethynyl-2’-deoxyuridine; Invitrogen, E10187) before the session, and perfused 3 hours (for OPC response analysis) or 24 hours (for glioma cell proliferation analysis) after the start of the stimulation. The effectiveness of optogenetic stimulation in the ChAT^+/wt^ x Ai230^flx/wt^ model was validated through cFos staining, as depicted in Figure S2. In Ai230 models, light delivery results in stimulation. Thus, we designated the group with the “laser on” as the stimulated cohort, while the non-stimulated group did not receive light (“mock”).

#### Terminal Field Stimulation of Cholinergic Axons

To stimulate cholinergic axon terminals in the thalamic and pontine DMG microenvironment, ChAT-Cre^+/+^ mice were injected unilaterally with either AAV-DJ-EF1a-DIO-hChR2 (E123A)::eYFP or AAV-DJ-EF1a-DIO-eYFP. The viral injections targeted the PPN or LDT, using the coordinates outlined above. A volume of 1 μL of the viral vector, with a titer of 4.3 × 10^12^ vg/ml (hChR2-eYFP) or 2.5 × 10^12^ vg/ml (-eYFP), was injected per site. Three weeks after viral injection, H3K27M-MADR cells (200.000 cells per mouse) were injected into either the thalamus or pons, as previously described. Three weeks later, optical ferrules were implanted into the glioma microenvironment, positioned 0.5 mm above the tumor cell injection site for each respective region. Following one week of recovery, optogenetic stimulation of cholinergic projections from the LDT or PPN was performed.

#### Optogenetic Stimulation of Local Cholinergic Projections

For optogenetic stimulation of local thalamic or pontine cholinergic neuronal cell bodies and their axonal projections in the DMG microenvironment, 4-week-old ChAT-Cre^+/+^ mice were injected with either AAV-DJ-EF1a-DIO-hChR2 (E123A)::eYFP (virus titer: 4.3 × 10^12^ vg/ml) or AAV-DJ-EF1a-DIO-eYFP (virus titer: 2.5 × 10^12^ vg/ml) into the thalamus or pons, positioned 0.5 mm above the tumor injection coordinates as described previously. Three weeks after viral expression, H3K27M-MADR cells (200,000 cells per mouse) were injected at the common thalamic or pontine coordinates. Three weeks later, optic ferrules were implanted at the viral injection site. One week after ferrule implantation, optogenetic stimulation was performed.

#### Optogenetic Inhibition of Midbrain Cholinergic Neurons

For optogenetic inhibition of midbrain cholinergic neurons, ChAT-Cre^+/+^ mice were injected with 1 μL of either AAV-Ef1a-DIO-NpHR3.0::eYFP or AAV-Ef1a-eYFP into the LDT or PPN. Following viral injection into the LDT, H3K27M-MADR cells were implanted into the thalamus, while for PPN-injected mice, pontine tumor cells were implanted. Optical ferrules were implanted three weeks after tumor implantation, with optogenetic inhibition performed one week later. For optogenetic inhibition, freely moving animals were connected to a 595 nm high-power LED system with a monofiber patch cord. NpHR-mediated inhibition of cholinergic neuron cell bodies was achieved by delivering 595 nm light at 1 Hz for 8 seconds every 10 seconds over a 30-minute period, with a light power output of 15 mW from the tip of the optic fiber. EdU was administered at the time of the optogenetic inhibition session, and tumor cell proliferation rate was assessed 24 hours later.

#### Fiber Photometry

Fiber photometry was employed across four experimental paradigms. For all recording sessions, mice were individually housed in an empty box and shielded from external noise and stimuli. Prior to each recording session, mice were allowed to habituate for 10 minutes after being connected to the patch cord. Data acquisition was performed using Synapse software in conjunction with an RZ5P lock-in amplifier (Tucker-Davis Technologies). GCaMP6m, GCaMP6s, and GRAB sensors were excited by frequency-modulated 465 and 405 nm LEDs (Doric Lenses). Optical signals were band-pass filtered through a fluorescence mini cube (Doric Lenses) and digitized at 6 kHz.

#### Fiber Photometry Recordings from Midbrain Cholinergic Neurons

For recordings from midbrain cholinergic neurons in the LDT and PPN, 4-week-old ChAT-Cre^+/+^ mice were intracranially injected with AAV-DJ EF1a-DIO-GCaMP6m (virus titer: 1.9 × 10^13^ vg/ml). Three weeks post-injection, an optical fiber (400 μm core diameter, NA = 0.48, Doric Lenses) was implanted above the injection site. In the same surgical procedure, H3K27M-MADR or control mTmG cells were injected into the thalamus or pons. One week after recovery, the first recording session was initiated, with recordings performed weekly for up to 4 weeks. Mice were recorded for 5 minutes per session.

#### Fiber Photometry Recordings of Acetylcholine Release in the DMG Microenvironment

For acetylcholine recordings in the glioma microenvironment, AAV-8-hSyn-DIO-GRAB_ACh3.0 (virus titer: 8.9 × 10^12^ vg/ml) was injected into 4-week-old ChAT-Cre^+/+^ mice, positioned 0.5 mm above the common thalamic or pontine tumor cell injection site. After viral expression, H3K27M-MADR or control mTmG cells were implanted into the pons or thalamus, with simultaneous optical fiber placement (400 μm core diameter, NA = 0.48, Doric Lenses) above the GRAB injection site. Recordings began one week after recovery and were performed weekly for up to 4 weeks. Mice were recorded for 10 minutes per session.

#### Fiber Photometry Recordings of DMG Cells During PPN/LDT Stimulation

To monitor calcium signals from DMG cells during optogenetic stimulation of midbrain cholinergic neurons in the LDT and PPN, we generated a DMG cell line (SU-DIPG6-GCaMP6s) lentivirally transduced with GCaMP6s (pLV-ef1-GCAMP6s-P2A-nls-tdTomato). 4-week-old NSG mice were retro-orbitally injected with AAV.PHP.eB-VAChTe1-(ChRmine::)eYFP, as previously described, to enable optogenetic stimulation of midbrain cholinergic neurons. Three weeks later, DMG cells were xenografted into the thalamus, with optical fiber placement (400 μm core diameter, NA = 0.48, Doric Lenses) above the implantation site and an additional fiber in the LDT or PPN for optogenetic stimulation. Recordings were initiated 6 weeks post-tumor xenograft. Mice were connected to both optogenetic and fiber photometry cables, with a 5-minute baseline recording followed by 5 minutes of optogenetic stimulation (‘laser on’) and an additional 5 minutes of recording after stimulation (‘laser off’).

In a separate cohort, we examined the role of CHRM1/CHRM3 antagonism on calcium signaling. In this experiment, single-shot optofluid cannulas (OsFC_400/430-0.66-Optic Fluid Cannula_3.5mm_FLT_100/170_0.0, Doric Lenses) were placed above the tumor implantation site. The cannulas, equipped with an optical fiber and metal tube, were connected to a Neurosyringe via a flexible tube. During ongoing recordings and optogenetic stimulation, the drug was delivered to the tumor cells with a volume of 2.5 μL.

#### Signal Processing and Data Analysis

Signal processing was performed using pMAT and custom MATLAB scripts (MathWorks). Raw fluorescence signals were debleached by fitting the data to either a mono-exponential or bi-exponential decay function. The resulting fluorescence traces were then Z-scored, where the Z-score for each time point was calculated relative to the baseline fluorescence. To visualize the Z-score data, a Locally Weighted Scatterplot Smoothing (LOESS) technique was applied to the time-series data. LOESS smoothing was performed with a smoothing fraction of 0.1 to generate a robust curve that effectively captures the underlying trends in the data while reducing noise. The area under the curve (AUC) was computed by integrating the Z-score signal over a defined time window following stimulus onset, using the trapezoidal rule to obtain a quantitative measure of the response magnitude.

#### Entrectinib Treatment Study for DMG Cell Spread Experiment

To investigate the effects of pan-TrkB inhibition on the spread of DMG cells in different anatomical compartments, a highly migratory patient-derived DMG culture (‘SU-DIPG13fl’) was xenografted into 4-week-old NSG mice. Cell suspensions were xenografted into the midbrain (n = 400.000 cells per mouse) using the following coordinates: AP=−3.3mm (from bregma), ML=+0.0mm, DV=−3.5mm. DMG cells were allowed to spread for a total of 12 weeks, with the start of treatment 11 weeks after xenografting. Mice were randomly assigned to treatment groups in a blinded fashion, with equal representation of male and female mice in each group. Mice were treated with entrectinib (HY-12678, MedChemExpress) at 120 mg/kg or vehicle controls (sterile H_2_O) orally daily for 7 consecutive days. Entrectinib was dissolved in 7% DMSO (Sigma), 10% Tween 80 (Sigma) in sterile H_2_O. Mice were perfused 24 hours after receiving the final dose of the drug.

#### Pharmaceutical Antagonism of Muscarinic Receptors M1 and M3

To assess a potential effect of muscarinic receptors M1 (CHRM1) and M3 (CHRM3) on the proliferative effect of cholinergic neuronal activity *in vivo*, ChAT-IRES-Cre^+/wt^ x Ai230^flx/wt^ mice were allografted with the MADR model as above and blind randomized to a treatment group. Four weeks after allograft implantation, mice were treated with local delivery to the DMG cells of the M1 receptor antagonist VU 0255035 (10 mg/kg; Tocris), the M3 receptor antagonist 4-DAMP (10 mg/kg; Tocris), or vehicle controls. Local delivery was performed as previously described, with implantation of an optofluidic cannula (iOFC_M3_320/430_4.0mm_PLGS, Doric Lenses) positioned 0.5 mm above the tumor injection site. Administration of with the drug or vehicle was done during the optogenetic stimulation session of each mouse. For immunohistological analysis of glioma cell proliferation, mice were perfused 24 hours after optogenetic stimulation.

#### Generation of DMG Cell Line with CHRM1 and CHRM3 Double Knockout

Patient-derived DMG cells (SU-DIPG17) were cultured in complete growth media at 37°C in a 5% CO_2_ incubator as described above. Cells were passaged at 80% confluency and maintained in optimal conditions. To generate a Cas9-expressing cell line, we used a lentiviral vector (pB-TRE3G-spCas9-Ubc-rtTA3-IRES-BlastR, Addgene #195506). For the generation of a Cas9-expressing DMG cell line (SU-DIPG17-Cas9), we used the inducible PiggyBac vector Pb-TRE3G-SpCas9-IRES-Blast (Addgene #195506), The transposition system for the inducible Cas9 vector was performed using the PiggyBac transposase system. DMG cells were transfected with 2.5 μg of the Pb-TRE3G-SpCas9-IRES-Blast vector and 0.5 μg of the PiggyBac transposase plasmid using FuGENE HD according to the manufacturer’s protocol. After 6 hours, the cells were provided with fresh media and allowed to recover for 6 days. Transfected cells were selected with 5 μg/mL blasticidin (Thermo Fisher Scientific). Successfully transduced cells, expressing both Cas9 and the blasticidin resistance marker, were expanded. Cas9 expression was confirmed by Western blotting.

For CRISPR-mediated knockout of CHRM1 and CHRM3, the CRISPR guide RNA (gRNA) plasmids were designed to target the coding exons of the CHRM1 (TGAGCAGGTACGTGGTATAG, chr11[62910838], Thermo Fisher Scientific) and CHRM3 (CGTTTGGCTCGGTACGTGAG, chr1[239907990], Thermo Fisher Scientific) genes using the TrueDesign Genome Editor (Invitrogen). DMG cells were passaged at 80% confluency and filtered into a single-cell suspension before electroporation to ensure optimal cell density. The cells (n=100.000) were electroporated with a total volume of 100uL OPTI-MEM (Gibco) containing gRNAs targeting CHRM1 and CHRM3. Electroporation was performed using the NEPA21 Electroporator (NEPA GENE) and the CU500 Cuvette Chamber (Bulldog Bio) under the following conditions: 300V pulse voltage, 50 ms pulse interval, 1 pulse, and a 5 ms pulse time. After electroporation, cells were immediately transferred into 2 mL of complete growth media and incubated at 37°C for 24 hours to recover. Twenty-four hours post-electroporation, the cells were selected with blasticidin (Thermo Fisher Scientific). After selection, knockout of CHRM1 and CHRM3 was confirmed by confocal microscopy of *in vitro* and *in vivo* cells.

#### Cerebral Slice Conditioned Media of LDT and PPN

ChAT-IRES-Cre^+/wt^ x Ai230^flx/wt^ mice aged 4 weeks (P28 to P30) were utilized to collect conditioned media from activated cholinergic neurons in either the LDT or PPN. Brief exposure to isoflurane induced unconsciousness in the mice before immediate decapitation. Extracted brains (cerebrum) were inverted and placed in an oxygenated sucrose cutting solution, then sliced at 300μm to target the region of the LDT or PPN. Slices (n=4 per mouse) were transferred to ACSF and allowed to recover for 30 minutes at 37°C, followed by an additional 30 minutes at room temperature. After recovery, the slices were transferred to fresh ACSF and positioned under a red-light LED using a microscope objective. The optogenetic stimulation paradigm consisted of 20-Hz pulses of red light for 30 seconds on, followed by 90 seconds off, repeated over a period of 30 minutes. Conditioned medium from the surrounding area was collected and stored frozen at −80°C. Stimulated slices were postfixed in 4% paraformaldehyde (PFA) for 30 minutes before cryoprotection in 30% sucrose solution for 48 hours. Successful stimulation of cholinergic neurons in each area was validated through cFos staining.

#### Slice Preparation for Electrophysiology

Coronal brain slices (300 μm) containing the LDT were prepared from 8-week-old mice of both sexes using a vibratome (Leica VT 1200S, Leica, Germany). All procedures followed a protocol approved by the Stanford University APLAC. Briefly, mice were anesthetized with isoflurane before brain extraction. Following decapitation, brains were rapidly removed and placed into ice-cold (0–4°C) artificial cerebrospinal fluid (ACSF) composed of (in mM): 110 choline chloride, 2.5 KCl, 1.2 Na_2_HPO_4_, 3.5 MgCl_2_, 0.5 CaCl_2_, 1.2 MgSO_4_, 10 glucose, and 26 NaHCO_3_, bubbled with 95% O_2_ and 5% CO_2_ to achieve a pH of 7.3 (osmolality: ~305 mOsm). Slices were then incubated for 15–20 minutes at 34°C in ACSF containing (in mM): 126 NaCl, 2.5 KCl, 1.2 Na_2_HPO_4_, 1 MgCl_2_, 1.5 CaCl_2_, 10 glucose, and 26 NaHCO_3_, with the pH adjusted to 7.3 (osmolality: ~305 mOsm). This step was followed by a one-hour incubation at room temperature (approximately 25°C) to allow for cellular equilibration before transferring to the recording chamber. All recordings were conducted at the physiologically optimal temperature of 34°C.

#### Electrophysiology

Brain slices were placed in a recording chamber mounted on an upright microscope (BX51WI, Scientifica, USA) and continuously perfused with oxygenated ACSF (95% O_2_ / 5% CO_2_) at a flow rate of 2–3 mL/min. All experiments were conducted at or near physiological temperature (33–34°C). The LDT was identified by locating the dorsal tegmental nucleus (DTN), which appears in the slice as a dark, bilateral, oval-shaped region below the fourth ventricle. The LDT is positioned laterally to the DTN (Figure S11C). Within the LDT oScarlet-positive cholinergic cells were identified and visualized using differential interference contrast (DIC) infrared optics with a sCMOS camera (Kinetix, Teledyne Vision Solutions©, USA). Spontaneous excitatory postsynaptic currents (sEPSCs) from cholinergic (oScarlet-positive) cells were recorded in whole-cell voltage-clamp mode at −70 mV, using borosilicate glass pipettes (5–8 MΩ) filled with a potassium-gluconate-based patch solution (in mM: 100 potassium gluconate, 20 KCl, 10 HEPES, 4 Mg-ATP, 0.3 Na-GTP, 10 Na-phosphocreatine, and 0.2 EGTA; pH adjusted to 7.3, osmolality 295 mOsm). sEPSC recordings were excluded if the holding currents exceeded −50 pA or if series resistance increased by more than 30 MΩ, which was continuously monitored. Field excitatory postsynaptic potentials (fEPSPs) were recorded using a glass pipette (1 MΩ) filled with 1 M NaCl, strategically positioned within the LDT region, where the highest density of cholinergic neurons (oScarlet +Ve) has been observed. This placement aimed to capture the maximum output potential in response to the injected input current. Presynaptic stimulation was accomplished using a concentric bipolar microelectrode (FHC Microelectrodes, USA), which was inserted approximately 20 μm in depth and about 100 μm dorsal to the LDT. Prior to each recording session, the location and intensity of the stimulus were adjusted to ensure that consistent inward currents of similar amplitude were evoked. Input currents were delivered through an ISO-Flex stimulus isolator (AMPI, IL), with precise control over the duration of the TTL pulse (a single square wave lasting 0.1 ms) generated by a Digidata 1550B, which was under the control of Clampex software. A series of pulses, ranging in intensity from 0 to 100 μA, were injected at 10-second intervals to measure the resulting output fEPSP in current clamp mode. To enhance clarity, stimulus artifacts from the field potential recordings were removed. All electrophysiological data were collected using a MultiClamp 700B amplifier, filtered at 2 kHz, digitized at 20 kHz by the Digidata 1550B, and analyzed with Clampex 11 software (Molecular Devices: Axon Instruments, USA).

#### EdU Incorporation Assay

EdU staining of glioma monocultures or glioma-neuron co-cultures was performed on glass coverslips in 96-well plates which were precoated with poly-l-lysine (Sigma) and laminin (Thermo Fisher Scientific). Neurosphere cultures were dissociated with TrypLE and plated onto coated coverslips with growth factor-depleted medium. Acetylcholine (0.5μM to 5μM, Tocris), VU 0255035 (10μM, Tocris), 4-DAMP (10μM, Tocris) and vehicle (DMSO) were added for specified times with 4 μM EdU. After 24 h the cells were fixed with 4% PFA in PBS for 20 min and then stained using the Click-iT EdU kit and protocol (Invitrogen) and mounted using Prolong Gold mounting medium (Life Technologies). Confocal images were acquired on a Zeiss LSM980 using Zen 2011 v8.1. Proliferation index was determined by quantifying the fraction of EdU-labelled cells divided by DAPI-labelled cells (monoculture experiments), or HNA-labeled cells (co-culture experiments) using confocal microscopy at 20× magnification. Per coverslip, 6 fields were imaged and quantified, including all DAPI^+^ or HNA^+^ cells within each field. The fields were selected systematically, with 3 fields from the center and 3 fields from the margins of the coverslip. Using this method, an average of 1000 cells per coverslip were counted. Quantification of images was performed by a blinded investigator.

#### Migration Assay

3D migration experiments were performed as previously introduced^83^ with some modifications. Briefly, 96-well flat-bottomed plates (Falcon) were coated with 2.5μg per 50μl laminin per well (Thermo Fisher) in sterile water. After coating, a total of 200μl of culture medium per well was added to each well. A total of 100μl of medium was taken from 96-well round bottom ULA plates containing ~200μm diameter neurospheres, and the remaining medium including neurospheres was transferred into the pre-coated plates. Images were then acquired using an Evos M5000 microscope (Thermo Fisher Scientific) at time zero, 24, 48, and 72 hours after encapsulation. Image analysis was performed using ImageJ by measuring the diameter of the invasive area. The extent of cell migration on the laminin was measured for all replicate wells normalized to the diameter of each spheroid at time zero and the data is presented as a mean ratio for three biological replicates.

#### Neuron-Glioma Co-Culture

For EdU incorporation assays, neurons were isolated from CD1 mice (The Jackson Laboratory) at P0 using the Neural Tissue Dissociation Kit Postnatal Neurons (Miltenyi), and followed by the Neuron Isolation Kit, Mouse (Miltenyi) per manufacturer’s instructions. After isolation, 200.000 neurons were plated onto circular glass coverslips (Electron Microscopy Services) pre-coated with poly-l-lysine (Sigma) and mouse laminin (Thermo Fisher Scientific). Neurons were cultured in BrainPhys neuronal medium containing B27 (Invitrogen), BDNF (10 ng ml—1, Shenandoah Biotech), GDNF (5 ng ml—1, Shenandoah Biotech), TRO19622 (5 μM; Tocris) and β-mercaptoethanol (Gibco). The medium was replenished on days *in vitro* (DIV) 1 and 3. On DIV 5, fresh medium was added containing 70,000 glioma cells and incubated for 48 h. After 48h incubation, EdU (10 μM) with or without the acetylcholine (0.5–5 μM, Tocris) was added and incubated for a further 24h. Following incubation, the cultures were fixed with 4% paraformaldehyde (PFA) for 20 min at room temperature and stained for immunofluorescence analysis. For EdU analysis, cells were stained using the Click-iT EdU Cell Proliferation kit (Thermo Fisher Scientific, C10337), before staining with primary antibodies mouse anti-human nuclei clone 235–1 (1:250; Millipore, MAB1281) and rabbit anti-microtubule-associated protein 2 (MAP2; 1:500, EMD Millipore, AB5622), overnight at 4 °C. For characterization of neuron subpopulations, cells were stained using mouse anti-VGLUT2 (1:500, Synaptic Systems, 135 421) and guinea pig anti-GAD65 (1:500, Synaptic Systems, 198 104). Following washing, slips were incubated in secondary antibodies, Alexa 488 donkey anti-mouse IgG (1:500, Jackson Immuno Research) and Alexa Fluor 555 donkey anti-rabbit (1:500, Invitrogen) and mounted using ProLong Gold Mounting medium (Life Technologies). Images were collected on a Zeiss LSM980 at 20× magnification, and proliferation index determined by quantifying percentage EdU-labelled glioma cells over total glioma cells (HNA immunopositivity to identify glioma cells). As described above, 6 fields were imaged and quantified per coverslip, including all HNA^+^ cells within each field. The fields were selected systematically, with 3 fields from the center and 3 fields from the margins of the coverslip. Using this method, an average of 1000 cells per coverslip were counted. To characterize the proportions of neuronal subpopulations, three randomly assigned coverslips were analyzed for VGLUT2 and GAD65 positivity, serving as markers for glutamatergic and GABAergic neurons, respectively. Every neuron on each coverslip was included in the analysis to calculate the proportions of the neuronal subpopulations.

#### Human iPSC-Derived Cholinergic Motor Neurons

Human induced pluripotent stem cells (iPSCs) of a 12-year-old male healthy donor (CW20032, Elixirgen) maintained under feeder-free conditions in a 96-well plate (20.000 cells per well) were rapidly differentiated to a cholinergic phenotype with Sendai virus mediated delivery of synthetic neurogenic transcription factor (CH-SeV, Elixirgen) as previously described.^84^ For each experiment, we plated the same number of iPSCs (n = 20.000) to ensure consistency. This number was determined after initial validation and optimization, during which we tested various conditions to identify the optimal environment for the successful differentiation of iPSCs into mature cholinergic motor neurons with the highest yield. The number of neurons in each well was counted on day 7 of the differentiation process to ensure consistent neuronal density across wells before adding glioma cells. Counting was performed using a 10x magnification. After successful morphological differentiation into cholinergic neurons at day 7, fresh medium (cholinergic neuron maintenance media, CH-MM, Elixirgen) was added containing 20.000 glioma cells (SU-DIPG17) and incubated for 48 h. After 48h incubation, EdU (4 μM) with or without VU 0255035 (10μM, Tocris) or 4-DAMP (10μM, Tocris) was added and incubated for a further 24h. Following incubation, the cultures were fixed with 4% paraformaldehyde (PFA) for 20 min at room temperature and further staining was processed as above described. Primary antibodies rabbit anti-Histone H3 (Mutated-K27M) (H3 K27M; 1:500, Abcam, ab190631), chicken anti-neurofilament-H (NF-H; 1:1000, AvesLabs, NFM), chicken anti-neurofilament-M (NF-M; 1:1000, AvesLabs, NFH), DAPI, and Click-iT EdU cell proliferation kit (Invitrogen, C10339 or C10337 were used. For synaptic puncta staining, neuron-glioma cultures were incubated for 72h. After fixation with 4% PFA, cholinergic neuron-to-glioma co-culture coverslips were incubated in blocking solution (3% normal donkey serum, 0.3% Triton X-100 in TBS) at room temperature for 1h. Primary antibodies chicken anti-neurofilament-H (NF-H; 1:1000, AvesLabs, NFM), chicken anti-neurofilament-M (NF-M; 1:1000, AvesLabs, NFH), guinea pig anti-vesicular acetylcholine transporter (VAChT; 1:500, Synaptic Systems, 139 105), mouse Anti-Histone H3 (tri methyl K27) (H3 K27M; 1:500, Abcam, ab6002), rabbit anti-cholinergic muscarinic receptor 1 (CHRM1; 1:500, Alomone Labs, AMR-001), and rabbit anti-cholinergic muscarinic receptor 3 (CHRM3; 1:500, Alomone Labs, AMR-006) were used in diluent (1% normal donkey serum in 0.3% Triton X-100 in TBS) and incubated at 4 °C overnight. Following washing, the coverslips were incubated in secondary antibody (Alexa 555 donkey anti-rabbit IgG, Invitrogen; Alexa 405 donkey anti-guinea pig IgG; Alexa 647 donkey anti-mouse IgG and Alexa 488 donkey anti-chicken IgG all used at 1:500, Jackson Immuno Research) overnight at 4 °C. Following washing, coverslips were mounted using ProLong Gold Mounting medium (Life Technologies). Images were collected on a Zeiss LSM980 confocal microscope using a 63× oil-immersion objective. Co-localization of synaptic puncta images were performed using a custom ImageJ (v.2.1.0/153c) processing script. In brief, the quantification determines co-localization of presynaptic VAChT and postsynaptic CHRM1/3 within a defined proximity of 1.5 μm. Background fluorescence is removed using rolling ball background subtraction and peaks detected using imglib2 DogDetection plugin which determines the region of interest for each channel. The percentage of total glioma ROIs that are within 1.5 μm of a neuron ROI is reported. The script was implemented in ImageJ (v.2.1.0/153c).

#### Optogenetic Stimulation of Cholinergic Neurons In Vitro

At day 7 of human iPSC differentiation, cholinergic motor neurons were transduced with either AAV-PHP.eB-VAChTe1-ChRmine:: eYFP or AAV-PHP.eB-VAChTe1-eYFP for 7 days. After successful viral transduction, glioma cells were added and cultured in optimized media as described above. The success of viral transduction was confirmed by eYFP expression visualized using fluorescent microscopy. On day 14 of human iPSC differentiation and 7 days after viral transduction, optogenetic stimulation was performed. Cholinergic neuron-glioma co-cultures were positioned under a red-light LED using a microscope objective. The optogenetic stimulation paradigm consisted of 20-Hz pulses of red light for 30 seconds on, followed by 90 seconds off, repeated over a period of 30 minutes. At the time of stimulation, EdU was added, and cells were fixed 24 hours after optogenetic stimulation.

#### Immunohistochemistry

All mice were anesthetized with intraperitoneal injections of 2.5% Avertin (tribromoethanol; Sigma-Aldrich, T48402), and transcardially perfused with 20 ml 0.1M phosphate buffer saline (PBS). Brains were postfixed in 4% paraformaldehyde (PFA) overnight at 4°C before cryoprotection in 30% sucrose solution for 48 hours. For sectioning, brains were embedded in optimum cutting temperature (OCT; Tissue-Tek) and sectioned coronally at 40 μm using a sliding microtome (Leica, HM450). For immunohistochemistry, brain sections were stained using the Click-iT EdU cell proliferation kit (Invitrogen, C10339 or C10337) according to manufacturer’s protocol. Tissue sections were then stained with antibodies following an incubation in blocking solution (3% normal donkey serum, 0.3% Triton X-100 in tris buffer saline) at room temperature for 30 minutes. Mouse anti-human nuclei clone 235–1 (1:100; Millipore, MAB1281), rabbit anti-ChAT (1:500; Abcam, ab223346), goat anti-Pdgfra (1:500; R&D Systems, AF1062), rat anti-MBP (1:200; Abcam, ab7349), chicken anti-mCherry (1:1000; Abcam, ab205402), guinea pig anti-Olig2 (1:500, synaptic systems, 292 015) or rabbit anti-cfos (1:500; Santa Cruz Biotechnology, sc-52) were diluted in 1% blocking solution (1% normal donkey serum in 0.3% Triton X-100 in TBS) and incubated overnight at 4°C. All antibodies have been validated in the literature for use in mouse immunohistochemistry. The following day, brain sections were rinsed three times in 1x TBS and incubated in secondary antibody solution for 2 hours at room temperature. All secondary antibodies were used at 1:500 concentration including Alexa 488 anti-rabbit, Alexa 488 anti-mouse, Alexa 488 anti-chicken, Alexa 594 anti-chicken, Alexa 647 anti-goat, Alexa 647 anti-rat, Alexa 647 anti-rabbit, Alexa 405 anti-guinea pig (all Jackson ImmunoResearch), and Alexa 555 anti-rabbit (Invitrogen). Sections were then rinsed three times in 1x TBS and mounted with ProLong Gold (Life Technologies, P36930).

#### Confocal Microscopy and Quantification

All image analysis were performed by experimenters blinded to the experimental conditions or genotype. Cell quantification within allografted or xenografted tumors was conducted by acquiring z-stacks using a Zeiss LSM980 scanning confocal microscope (Carl Zeiss). A 1-in-6 series of coronal brain sections were selected, with 4 slices (40μm thickness) analyzed in the grafted brain area (thalamus, or pons). Brain tissue damaged during perfusion or tissue processing was excluded from histological analysis. Tumor cells were identified as GFP^+^ (MADR allografts) or HNA^+^ (patient-derived xenografts) and quantified in each field to determine the proliferation index, calculated as the percentage of GFP^+^ cells co-labeled with EdU. Quantification was performed using a 20x magnification field, with 6 fields selected from each of 4 brain slices per animal, in which all cells were quantified. The selection of the 6 fields was done systematically, covering both the tumor core and the tumor margin equally, with the needle injection site serving as the starting point. On average, this quantification method resulted in counting approximately 500 cells per animal. OPCs were identified by PDGFRa staining and quantified as the percentage of PDGFR^+^ cell co-labeled with EdU.

#### 3D Rendering

Images shown in Figure 4D and Figures S7A and S7B were captured by acquiring Airyscan z-stacks using a Zeiss LSM980 scanning confocal microscope. Images were taken with 0.15 μm z-steps at a resolution of 1024×1024 pixels and a 63x magnification field with oil immersion. Following Airyscan image processing, Imaris was used to create 3D volume surface renderings of each z-stack. Raw, unprocessed data images are provided in the raw data files in the supplement.

#### Western Blotting

For western blot analysis of NLGN3 in conditioned media, samples were prepared by adding LDS buffer and β-mercaptoethanol, heated, and loaded onto Tris-acetate gels. After electrophoresis and transfer onto PVDF membranes, the membranes were blocked and incubated with primary antibodies against the NLGN3 ectodomain (Abcam #ab192880). Following incubation with secondary antibody and washing, the membranes were developed using chemiluminescent substrate (Thermo Scientific #34580).

#### Measurement of BDNF and Acetylcholine in Conditioned Media

The concentration of BDNF (R&D Systems #DBNT00) and acetylcholine (Sigma-Aldrich # MAK056) in conditioned media was measured using ELISA. We compared the concentrations of conditioned media obtained from non-stimulated, LDT-stimulated, and PPN-stimulated brain slices as described above. Protocols were performed according to the manufacturer’s instructions. The optical density was measured at 450 nm with an absorbance microplate reader (Molecular Devices, SpectraMax M3). The total concentrations were determined as pg/mL from the standard curve. To confirm the specificity of the respective kit, medium and lysis buffer without protein extract were used as negative controls.

#### Single-Cell RNA Sequencing Analysis of Published Data

##### Cell filtering

We excluded cells with a low number of detected genes, using a cutoff of 2000 genes for smart-seq2 data and a cutoff of 1000 genes for the other types of sequencing data.

##### Gene filtering

In analyses that necessitated gene filtering, we kept the 7000 genes with the highest mean expression across cells.

##### Normalization

UMI counts were converted to counts per million (CPM). Each entry in the expression matrix was then normalized according to $E\;=\;log_{2}\left(\frac{CPM}{10}\;+\;1\right)$. The same normalization was used for transcripts per million (TPM) values. The values were divided by 10 as the actual complexity is assumed to be in the realm of about 100,000 and not 1 million as implied by the CPM and TPM measures.

##### Centering

To illustrate intra-tumor heterogeneity in gene expression (Figure S9A), centering was performed individually for each sample. This involved subtracting each gene’s mean expression value across all cells within the sample. Cells within each sample were then sorted based on their scores for the cholinergic genes using the sigScores function available at https://github.com/jlaffy/scalop.

For the pseudo-bulk data analysis (Figure S9B), expression values were first averaged across malignant cells within each sample. Subsequently, log-normalization was applied, but no centering was performed.

##### Correlation with cell states

Utilizing the OPC-like signature outlined by Neftel et al.,^46^ we conducted the following analyses for both H3K27M DGM samples and medulloblastoma samples:

- We computed the correlations within each sample between the cells’ OPC-score and the expression values of each selected cholinergic gene.
- We calculated the correlations across samples within each study, between the pseudo-bulk sample OPC scores and the pseudo-bulk gene expression levels.

These correlation values were then averaged across samples and studies, respectively (see Figure 5A–5C).

Additionally, we assessed correlations across samples in other tumor types using more cell-state signatures (Neftel et al., 2019^46^; Gavish et al., 2023^49^) (Figure S9C). Significant correlations (P < 0.05 after FDR correction) were considered.

##### Lineage vs stemness analysis

Focusing on the H3K27M DGM data published in the Filbin et al.^15^ study, we computed a ‘stemness score’ for each cell. This score is determined by subtracting either the cell’s OC score or its AC score from its OPC score, whichever is higher (the maximum of the two). We also calculated a ‘lineage score’ for each cell, defined as the maximum between the OC and AC scores. In cases where the AC score is higher, it is multiplied by −1. If both AC and OC scores are negative, a 0 value is assigned with some jitter. Additionally, we identified cells with a centered CHRM1 value > 1 and cells with a centered CHRM3 value > 1. All scoring and centering procedures were performed per sample.

### QUANTIFICATION AND STATISTICAL ANALYSIS

To determine the appropriate sample size for *in vivo* proliferation studies, a power analysis was conducted using an unpaired t-test to compare the means of two independent groups. The significance level was set to 0.05 (two-tailed), with a target power of 0.8. Based on pilot data for our allograft and xenograft models in combination with optogenetic stimulation, the power analysis indicated that a sample size of 4 animals per group would be required to achieve 80% power. The achieved power for this sample size was calculated to be 0.939. Gaussian distribution was confirmed using the Shapiro-Wilk test. Parametric data were analyzed with unpaired two-tailed Welch’s t-tests or one-way ANOVA with Tukey’s post hoc tests. Significance was set at p < 0.05. The used statistical test is indicated in the figure legend. GraphPad Prism 10 was used for statistical analyses and data illustrations.

## Supplementary Material

Table S1

Table S2

Source Data

4

Supplemental information can be found online at https://doi.org/10.1016/j.cell.2025.05.031.

## Figures and Tables

**Table:** KEY RESOURCES TABLE

REAGENT or RESOURCE	SOURCE	IDENTIFIER
Antibodies		
Anti-rabbit cFos	Santa Cruz Biotechnology	sc-52
Anti-rabbit choline acetyltransferase	Abcam	ab223346
Anti-rabbit choline acetyltransferase	Abcam	ab181023
Anti-mouse human nuclear antigen 235-1	Millipore	MAB1281
Anti-rabbit red fluorescent protein	Rockland	600-401-379
Anti-goat PDGFRa	R&D systems	AF1062
Anti-chicken mCherry	Abcam	ab205402
Anti-rat myelin basic protein	Abcam	ab7349
Anti-mouse nestin	Abcam	ab6320
Anti-neuroligin 3	Abcam	ab192880
Anti-rabbit CHRM1	Alomone Labs	AMR-001
Anti-rabbit CHRM3	Alomone Labs	AMR-006
Anti-guinea pig Olig2	Synaptic Systems	292 015
Anti-rabbit H3 K27M	Abcam	ab190631
Anti-mouse H3 K27M	Abcam	ab6002
Anti-chicken neurofilament-H	AvesLabs	NFH
Anti-chicken neurofilament-M	AvesLabs	NFM
Anti-guinea pig vAChT	Synaptic Systems	139 105
Anti-rat tdTomato	Kerafast	EST203
Anti-rabbit GFP	Thermo Fisher Scientific	A11122
Alexa Fluor 405 donkey anti-guinea pig	Jackson ImmunoResearch	706-475-148
Alexa Fluor 488 donkey anti-rabbit	Jackson ImmunoResearch	711-545-152
Alexa Fluor 488 donkey anti-mouse	Jackson ImmunoResearch	715-545-150
Alexa Fluor 488 donkey anti-chicken	Jackson ImmunoResearch	703-545-155
Alexa Fluor 555 donkey anti-rabbit	Invitrogen	A-31572
Alexa Fluor 594 donkey anti-chicken	Jackson ImmunoResearch	703-585-155
Alexa Fluor 647 donkey anti-goat	Jackson ImmunoResearch	705-605-147
Alexa Fluor 647 donkey anti-rabbit	Jackson ImmunoResearch	711-605-152
Alexa Fluor 647 donkey anti-rat	Jackson ImmunoResearch	712-605-150
Bacterial and virus strains		
AAV-Ef1a-DIO-NpHR3.0::eYFP	Stanford University Gene Vector and Virus Core	GVVC-AAV-58
AAV-Ef1a-DIO-eYFP	Stanford University Gene Vector and Virus Core	GVVC-AAV-13
AAV-8-hSyn-DIO-GRAB_ACh3.0	Stanford University Gene Vector and Virus Core	GVVC-AAV-239
AAV-DJ EF1a-DIO-GCaMP 6m	Stanford University Gene Vector and Virus Core	GVVC-AAV-92
AAV-DJ-EF1a-DIO hChR2 (E123A)-eYFP	Stanford University Gene Vector and Virus Core	GVVC-AAV-25
AAV.PHPeB-VAChTe1-ChRmine::eYFP	This paper	N/A
For AAV.PHP.eb-eHGT_78h-Cre-PEST	This paper	Addgene: 231791
TIT2L-XCaMPG-ICL-ChRmine-TS-oScarlet-Kv2.1-IRES2-tTA2	This paper	Addgene: 234865
CHRM1 - TGAGCAGGTACGTGGTATAG	Thermo Fisher Scientific	N/A
CHRM3 - CGTTTGGCTCGGTACGTGAG	Thermo Fisher Scientific	N/A
Pb-TRE3G-SpCas9-IRES-Blast	Addgene	195506
Biological samples		
Human induced pluripotent stem cells	Elixirgen	CW20032
Chemicals, peptides, and recombinant proteins		
DMEM	Thermo Fisher Scientific	11320082
Neurobasal-A	Invitrogen	10888-022
B27-A	Thermo Fisher Scientific	12587-010
Heparin solution	STEMCELL Technologies	07980
Human bFGF	Shenandoah Biotech	100-146
Human bEGF	Shenandoah Biotech	100-26
Human PDGF-AA	Shenandoah Biotech	100-16
Human PDGF-BB	Shenandoah Biotech	100-18
GDNF	Shenandoah Biotech	200-37
BDNF	Shenandoah Biotech	100-01
Insulin	Sigma-Aldrich	I9278
2-Mercaptoethanol	Sigma-Aldrich	63689
TrypLE Express Enzyme	Invitrogen	12604013
Laminin Mouse Protein	Thermo Fisher Scientific	23017015
Poly-L-Lysine solution	Sigma-Aldrich	P4707
Acetylcholine	Tocris	2809
VU0255035	Tocris	3727
4-DAMP	Tocris	0482
Neurexin	R&D systems	5268-NX
ANA-12	Milipore Sigma	SML0209
Scopolamine hydrobromide	Tocris	1414
Mecamylamine hydrochloride	Tocris	2843
Tetrodoxin citrate	Tocris	1069
Picrotoxin	Tocris	1128
NBQX	Tocris	0373
Entrectinib	MedChemExpress	HY-12678
Perampanel	Cayman Chemical Company	23003
DMSO	Sigma-Aldrich	D2650
Molecular Probes ProLong Gold Antifade Mountant	Thermo Fisher Scientific	P36930
BrainPhys Neuronal medium	STEMCELL Technologies	05790
TRO19622	Tocris	2906
Hibernate-A	Thermo Fisher Scientific	A12475-01
HEPES-HBSS with DNase	Worthington Biochemical	LS002007
Liberase	Roche Applied Sciences	05401054001
Glutamax	Invitrogen	35050-061
Sodium pyruvate	Invitrogen	11360070
MEM non-essential amino acids	Thermo Fisher Scientific	11140076
Antibiotic-antimycotic	Thermo Fisher Scientific	15240096
N21-MAX	Sigma-Aldrich	A9165
Trace elements B	Corning	25-022-Cl
N-acetyl cysteine	Sigma-Aldrich	A9165
CNTF	PeproTech	450-13
NT-3	PeproTech	450-03
Tribromoethanol	Sigma-Aldrich	T48402
5-ethynyl-2’-deoxyuridine	Invitrogen	E10187
Critical commercial assays		
Click-iT EdU cell proliferation kit	Invitrogen	C10339
Neural Tissue Dissociation Kit Postnatal Neurons	Miltenyi Biotec	130-094-802
Neuron Isolation Kit, Mouse	Miltenyi Biotec	130-115-390
Total BDNF ELISA Kit	R&D Systems	DBNT00
Choline/Acetylcholine Quantification Kit	Sigma-Aldrich	MAK056
Experimental models: Cell lines		
SU-DIPG13fl	Stanford Medical Center	N/A
SU-DIPG17	Stanford Medical Center	N/A
SU-DIPG19	Stanford Medical Center	N/A
SU-DIPG92	Stanford Medical Center	N/A
SU-DIPG6-GCaMP6s	Stanford Medical Center, modified for this paper	N/A
SU-DIPG17 CHRM1/3-KO	Stanford Medical Center, modified for this paper	N/A
H3K27M-MADR	This paper	N/A
mTmG	This paper	N/A
Experimental models: Organisms/strains		
Mouse: B6;129S6-Igs7<tm230(tetO-XCaMPG, CAG-ChRmine*/oScarlet*,-tTA2)Daigl>/J	This paper	The Jackson Laboratory: 037944
Mouse: ChAT-IRES-Cre	The Jackson Laboratory	006410
Mouse: NOD-SCID-IL2R-gamma chain-deficient	The Jackson Laboratory	005557
Mouse: C57BL/6J	The Jackson Laboratory	000664
Software and algorithms		
Image J2 V2.14.0/1.54f	Fiji	N/A
GraphPad Prism 10.2.2 (341)	GraphPad Software	N/A
Adobe Illustrator 2024 V28.5	Adobe	N/A
Imaris V10.0.0	Oxford Instruments	N/A
Synapse	Tucker-Davis Techologies	N/A
MATLAB R2021b	MathWorks	https://www.mathworks.com/products/matlab.html
pMAT v1-2	Barker Lab	https://github.com/djamesbarker/pMAT
Other		
Fiber Optic Rotary Joint Patch Cables (Optogenetics)	Thorlabs	RJPSL2
Mono Fiberoptic Cannula_(Optogenetics)	Doric Lenses Inc.	MFC_200/240-0.22_4.5mm_MF2.5_FLT
Optofluid Cannula with interchangeable injectors (Optogenetics)	Doric Lenses Inc.	iOFC_M3_250/350_4.0mm_PLGG
Mono Fiberoptic Patchcord (Fiber photometry)	Doric Lenses Inc.	MFP_400/430/1100-0.57_1m_FCM_CM3_LAF
Mono Fiberoptic Cannula (Fiber photometry)	Doric Lenses Inc.	MFC_400/430-0.66_4mm_MF2.5_FLT
Single-Shot Optic Fluid Cannula (Fiber photometry)	Doric Lenses Inc.	OsFC_400/430-0.66Optic Fluid Cannula_3.5mm_FLT_100/1700.0

## Highlights

- Midbrain cholinergic activity increases OPC and DMG proliferation
- Effects of cholinergic long-range projections on OPCs and DMGs are circuit dependent
- Reciprocally, DMG increases the activity of midbrain cholinergic neurons
- The muscarinic receptors M1 and M3 are identified as therapeutic targets in DMG
