# Supplementary material for: Cholinergic neuronal activity promotes diffuse midline glioma growth through muscarinic signaling
Source: Cell. Author manuscript; Available in PMC 2025 Aug 29. (PMC12396346; doi:10.1016/j.cell.2025.05.031)
Supplement: 4 [file NIHMS2096866-supplement-4.pdf]

Supplemental figures

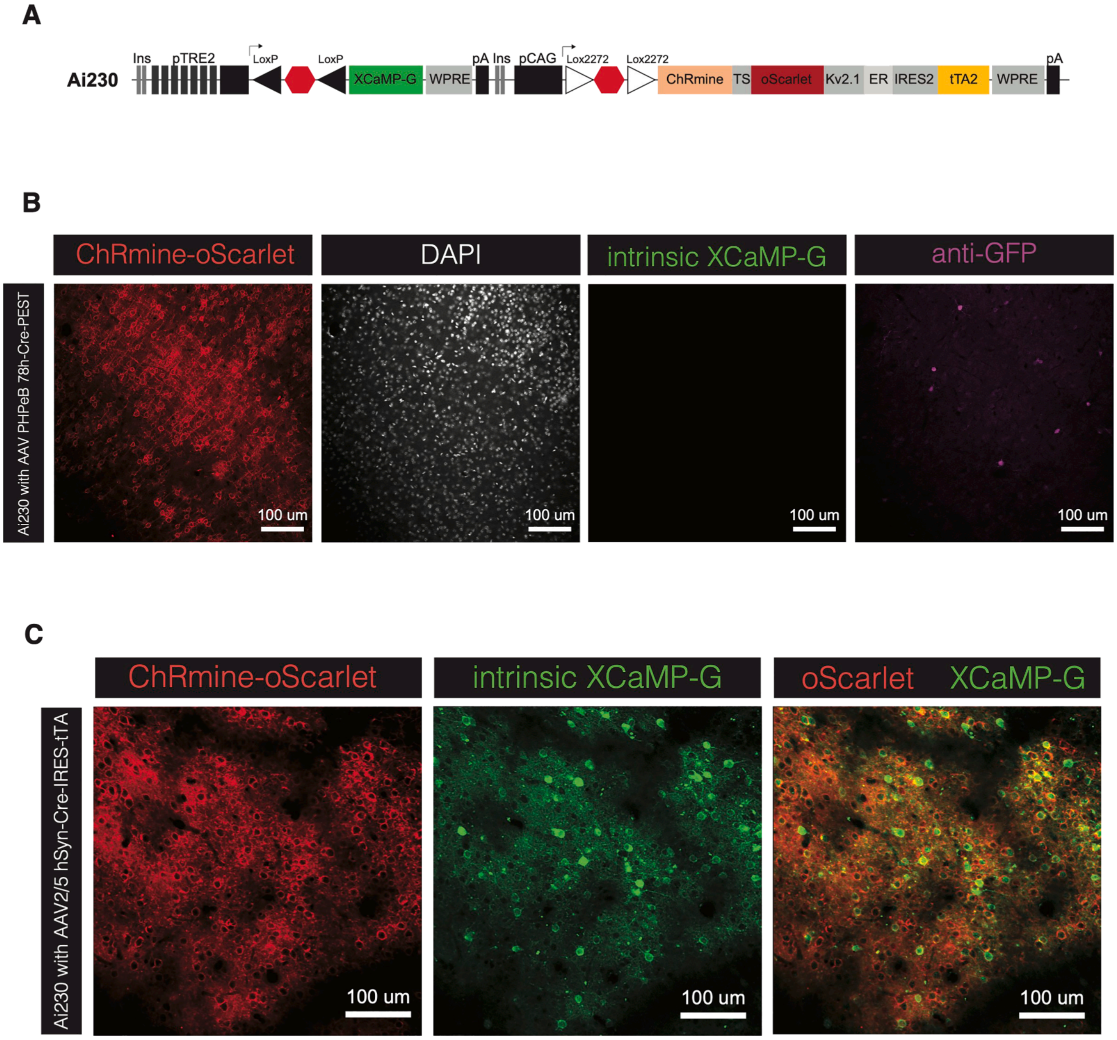

Figure S1. Validation of the TIGRE2.0 transgenic reporter line Ai230, related to Figure 1

(A) Schematic of the Ai230 reporter transgene in the TIGRE locus.

(B) Confocal images of coronal sections from the posterior cortex of Ai230 mice systemically injected with an AAV driving Cre under the excitatory enhancer eHGT_078h.[75] Cre delivery induced ChRmine-oScarlet expression, but no intrinsic XCaMP-G labeling was detected. The minimal XCaMP-G expression in Ai230 upon Cre delivery is likely due to low tTA2 levels in this construct. Sparsely XCaMP-G-labeled cells could be detected only after GFP-antibody staining and with the use of unusually high PMT gain and laser power.

(C) Two-photon microscopy images through a cranial window above the posterior cortex of Ai230 mice injected with a bicistronic AAV driving Cre and tTA. In contrast to Cre-delivery only, the additional delivery of tTA enabled XCaMP-G expression in Ai230. Thus, Ai230 serves as a Cre-dependent reporter for ChRmine-oScarlet, with the capacity for dual ChRmine-oScarlet/XCaMP-G reporting in the presence of Cre and tTA.

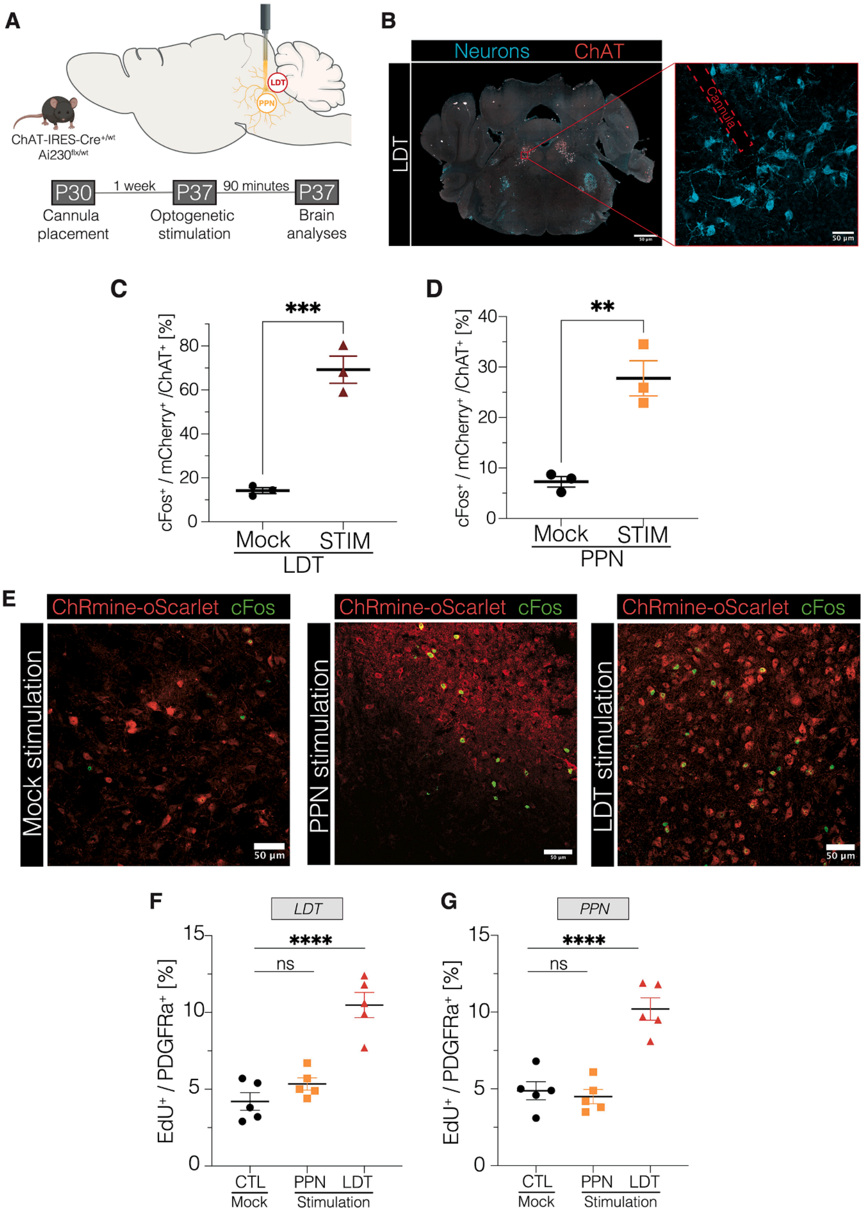

Figure S2. Validation of experimental paradigms, related to Figure 1

(A) Schematic of experimental paradigm for validation of optogenetic stimulation of cholinergic neurons in either LDT or PPN.

(B) Confocal micrographs showing cannula placement in the LDT. All neurons labeled by mCherry: turquoise, cholinergic neurons labeled by ChAT: red, scale bars, 50 μm.

(C) Quantification of activated cholinergic neurons (cFos$^+$/mCherry$^+$/ChAT$^+$) after optogenetic stimulation of LDT (CTL, LDT, n = 3 mice). Unpaired two-tailed Welch's t test; ***$p < 0.001$.

Data = mean ± SEM.

(D) Quantification of activated cholinergic neurons (cFos+/mCherry+/ChAT+) after optogenetic stimulation of PPN (CTL, PPN, n = 3 mice). Unpaired two-tailed Welch's t test; **$p < 0.01$.

Data = mean ± SEM.

(E) Confocal micrographs showing cFos staining in cholinergic neurons. ChRmine-oScarlet: red, cFos: green, scale bars, 50 μm.

(F and G) OPC response in (F) LDT, and (G) PPN after optogenetic stimulation of cholinergic neurons (CTL, PPN, and LDT, n = 5 mice/group). One-way analysis of variance (ANOVA) with Tukey's post hoc analysis; ****$p < 0.0001$, ns: non-significant. Data = mean ± SEM.

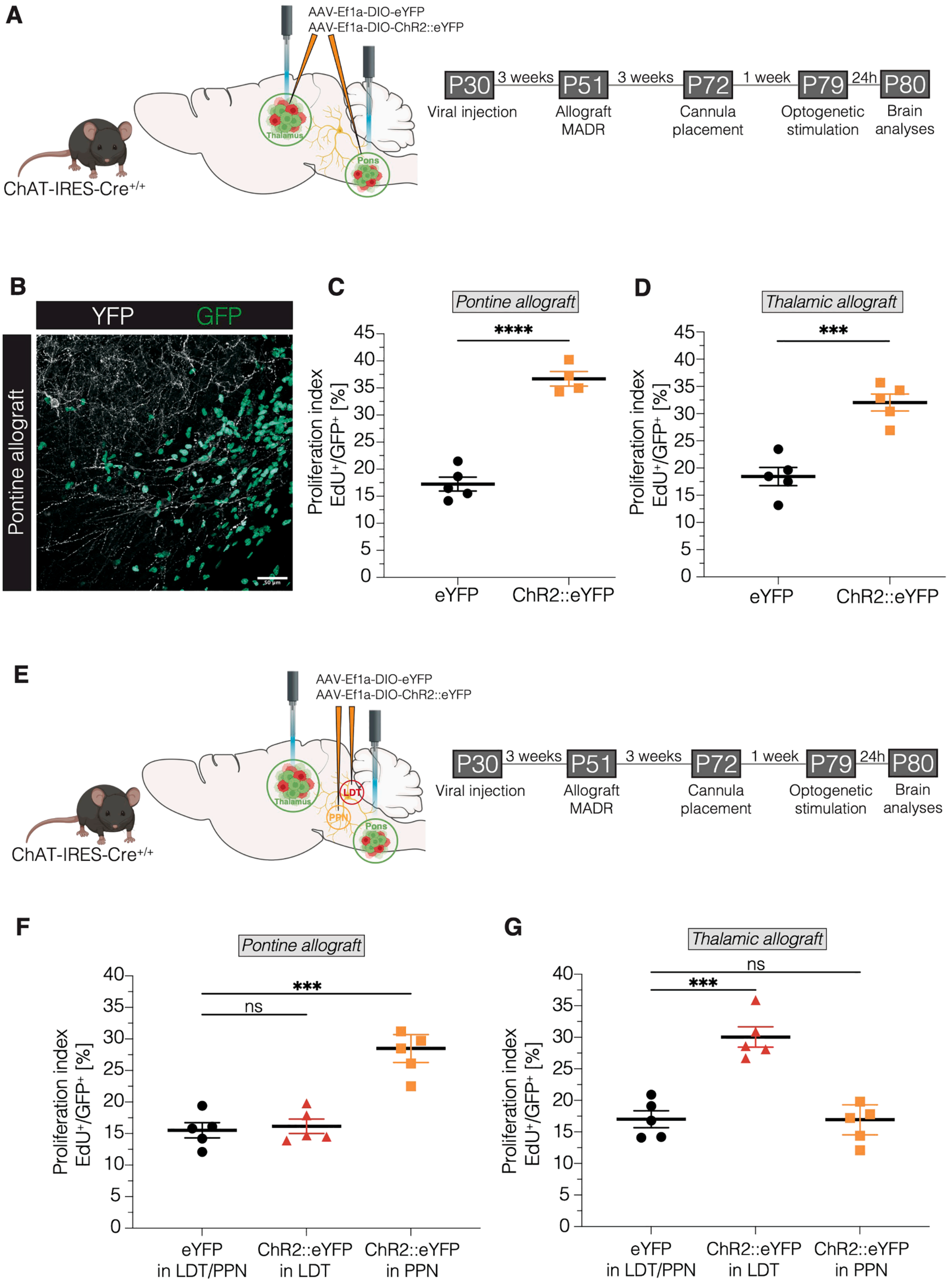

Figure S3. Stimulation of local and long-range cholinergic projections increase DMG cell proliferation, related to Figure 2

(A) Schematic of the experimental paradigm for stimulating local cholinergic projections from thalamic and pontine allografts.

(B) Confocal micrograph showing YFP expression in local cholinergic projections in the peritumoral microenvironment of a pontine allograft. GFP: green, YFP: white, scale bar, 50 μm.

(legend continued on next page)

(C and D) Proliferation index (EdU+/GFP+) of (C) pontine and (D) thalamic allografts in mice optogenetically stimulated of the peritumoral cholinergic projections. “Pontine allograft-eYFP,” “Thalamic allograft-eYFP,” and “Thalamic allograft-ChR2::eYFP” $n = 5$ mice/group; “Pontine allograft-ChR2::eYFP” $n = 4$ mice/group. Unpaired two-tailed Welch’s t test; ****$p < 0.0001$, ***$p < 0.001$. Data = mean ± SEM.

(E) Schematic of the experimental paradigm for terminal field stimulation of cholinergic projection from either LDT or PPN to thalamic or pontine allografts.

(F and G) Proliferation index (EdU+/GFP+) of (F) pontine and (G) thalamic allografts in mice with terminal field stimulation of either LDT or PPN projections. “Pontine allograft-eYFP,” “Pontine allograft-ChR2::eYFP in LDT,” “Pontine allograft-ChR2::eYFP in PPN,” “Thalamic allograft-ChR2::eYFP in LDT,” and “Thalamic allograft-ChR2::eYFP in PPN” $n = 5$ mice/group; “Thalamic allograft-eYFP” $n = 4$ mice/group. One-way analysis of variance (ANOVA) with Tukey’s post hoc analysis; ***$p < 0.001$, ns = non-significant. Data = mean ± SEM.

CellPress
OPEN ACCESS

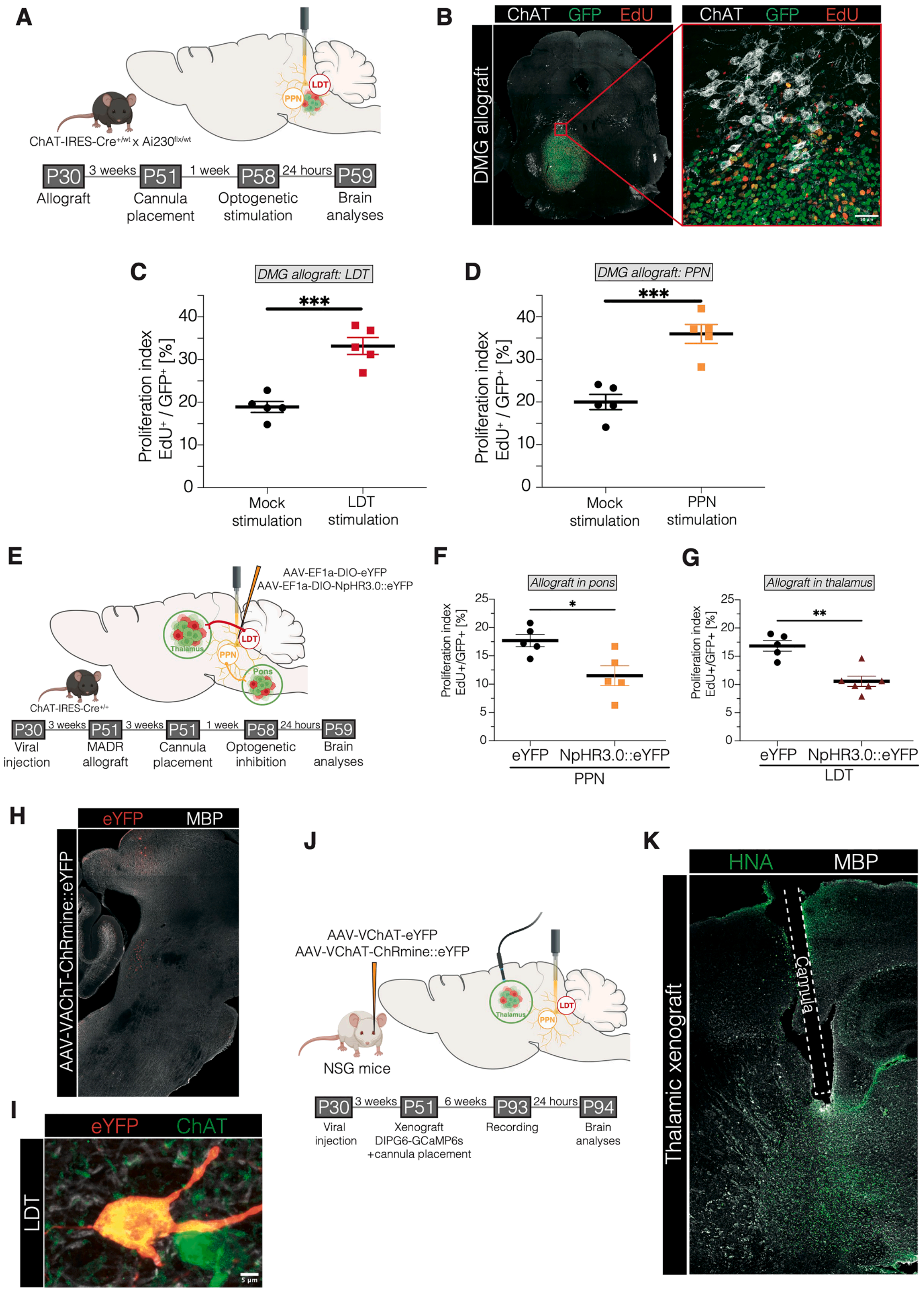

Figure S4. Cholinergic activity in the PPN and LDT promotes DMG cell proliferation, related to Figure 2

(A) Schematic of the experimental paradigm for optogenetic stimulation of the LDT and PPN with locally allografted DMG cells into each nucleus.

(B) Confocal micrographs showing DMG cells in cholinergic midbrain nuclei. GFP: green, EdU: red, ChAT: white; scale bar, 50 μm.

(C and D) Proliferation index (EdU+/GFP+) of (C) LDT and (D) PPN allografts in mice with either mock-, LDT-, or PPN-stimulation (*n* = 5 mice/group). Unpaired two-tailed Welch's t test; ****p* < 0.001. Data = mean ± SEM.

(E) Schematic of the experimental paradigm for optogenetic inhibition of cholinergic neurons of the LDT and PPN with pontine or thalamic allografts.

(legend continued on next page)

(F and G) Proliferation index (EdU+/GFP+) of (F) pontine and (G) thalamic allografts in mice with either -YFP or -NpHR3.0::YFP inhibition (n = 5 mice/group). Unpaired two-tailed Welch's t test; $^{**}p < 0.01$, $^{*}p < 0.05$. Data = mean ± SEM.

(H) Confocal micrograph showing YFP expression (from AAV.PHP.eB-VAChTe1-ChRmine::eYFP) in the brainstem in the expected distribution of cholinergic nuclei. YFP: red, MBP: white.

(I) High-magnification confocal micrograph showing YFP expression (from AAV.PHP.eB-VAChTe1-ChRmine::eYFP) co-localized with ChAT in a cholinergic neuron in the LDT. YFP is shown in red, ChAT in green, scale bar, 5 μm.

(J) Schematic of the experimental paradigm for fiber photometry recordings of calcium activity in thalamic xenografts.

(K) Confocal micrograph showing the fiber photometry cannula placement within the thalamic xenograft. HNA: green, MBP: white.

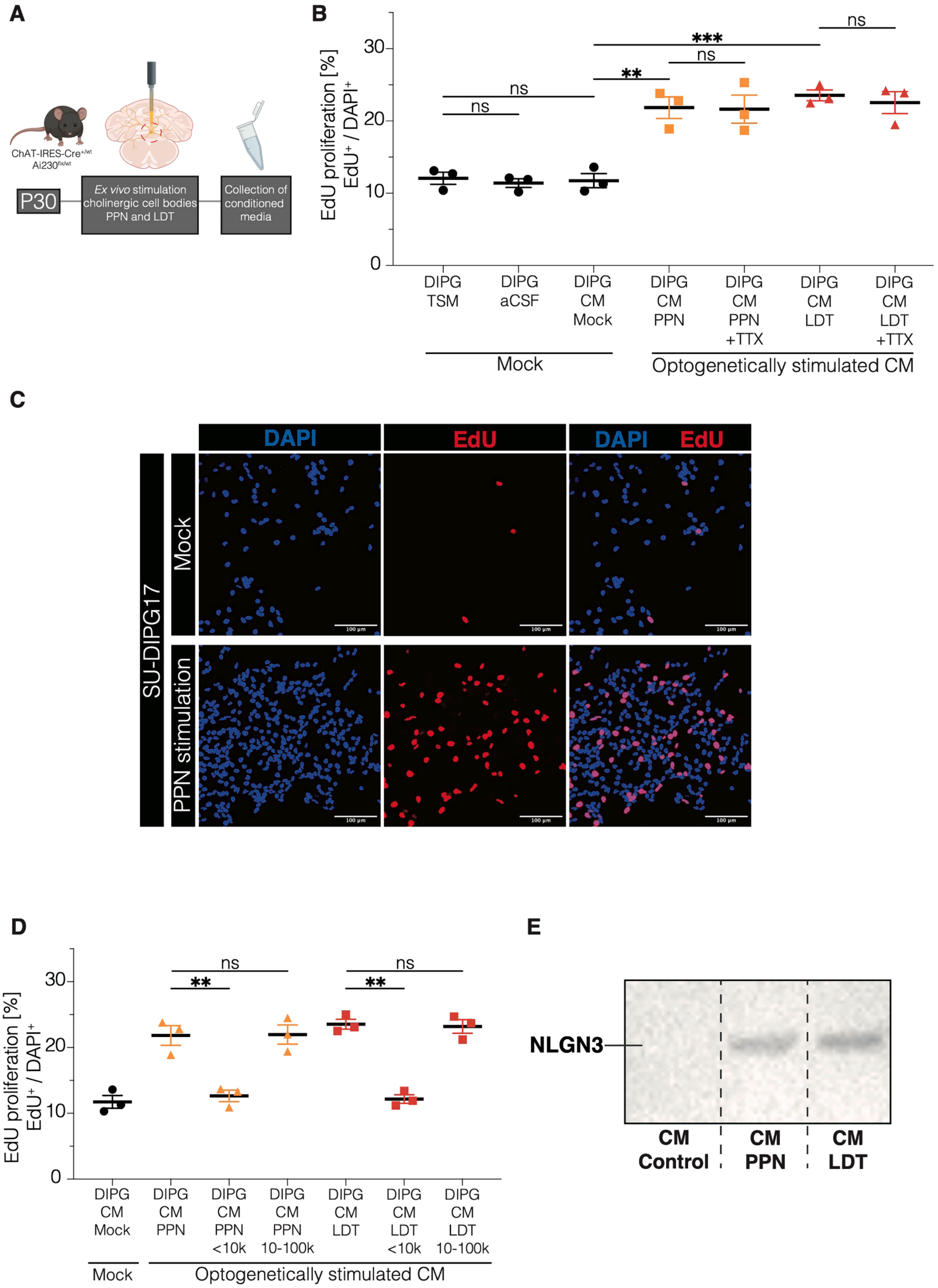

Figure S5. Midbrain cholinergic neurons promote DMG growth in part through paracrine factors, related to Figure 3

(A) Schematic of experimental paradigm for collection of conditioned media (CM) after *ex vivo* optogenetic stimulation of cholinergic neuronal cell bodies within the LDT or PPN in midbrain explants of 4-week-old ChAT-IRES-Cre+/wt x Ai230flx/wt mice.

(B) Quantification of DMG cell proliferation (EdU+/DAPI+) when adding CM after *ex vivo* stimulation of LDT or PPN compared with CM from mock-stimulated slices. One-way analysis of variance (ANOVA) with Tukey's post hoc analysis; $^{**}p < 0.01$, $^{***}p < 0.001$, ns: non-significant.

(legend continued on next page)

Data = mean ± SEM; n = three independent experiments, each with three wells per condition; each data point represents the mean of three wells per condition for a given experiment.
(C) Confocal micrographs showing EdU-labeled DMG proliferation after adding CM from mock-stimulated slices (upper images) and from PPN stimulation (bottom images). DAPI: blue, EdU: red, scale bars, 100 μm.
(D) Proliferation index (EdU+/DAPI+) after fractionation of the CM by molecular weight. One-way ANOVA with Tukey's post hoc analysis; $^{**}p < 0.01$, ns: non-significant. Data = mean ± SEM; n = three independent experiments, each with three wells per condition; each data point represents the mean of three wells per condition for a given experiment.
(E) Western blot analysis of NLGN3 in CM from non-stimulated midbrain explants ("Control") as well as CM from optogenetically stimulated PPN explants and from LDT explants.

CellPress
OPEN ACCESS

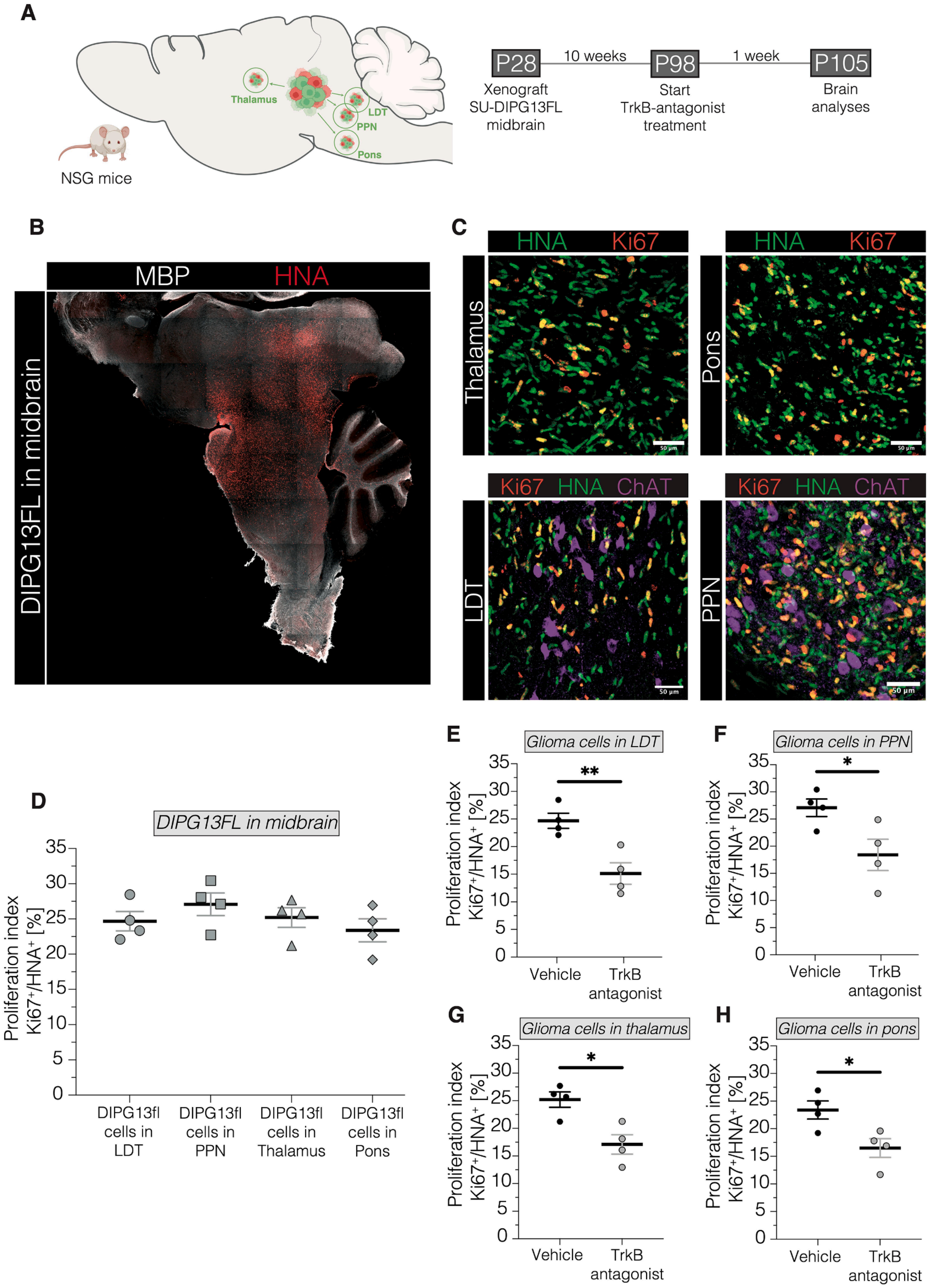

Figure S6. Disruption of BDNF-TrkB signaling in DMG cells is effective across different anatomical compartments, related to Figure 3

(A) Schematic of the experimental paradigm for examining the effectiveness of a BDNF-TrkB antagonist (entrectinib, administered orally) against xenografted DMG located in various anatomical compartments.

(legend continued on next page)

(B) Representative confocal micrograph showing a sagittal brain section illustrating the migratory spread of the DMG cell line across different anatomical compartments. HNA: red, MBP: white.

(C) Confocal micrographs showing HNA-labeled DMG cells in various anatomical regions. HNA: green, Ki67: red, ChAT: violet. Scale bars, 50 μm.

(D–H) Quantification of the proliferation index (Ki67+/HNA+) of DMG cells in (D) all analyzed anatomical areas, and in mice treated with either TrkB antagonist or vehicle control in (E) LDT, (F) PPN, (G) thalamus, and (H) pons (n = 4 mice/group). Unpaired two-tailed Welch's t test; $^{**}p < 0.01$, $^{*}p < 0.05$. Data = mean ± SEM.

CellPress
OPEN ACCESS

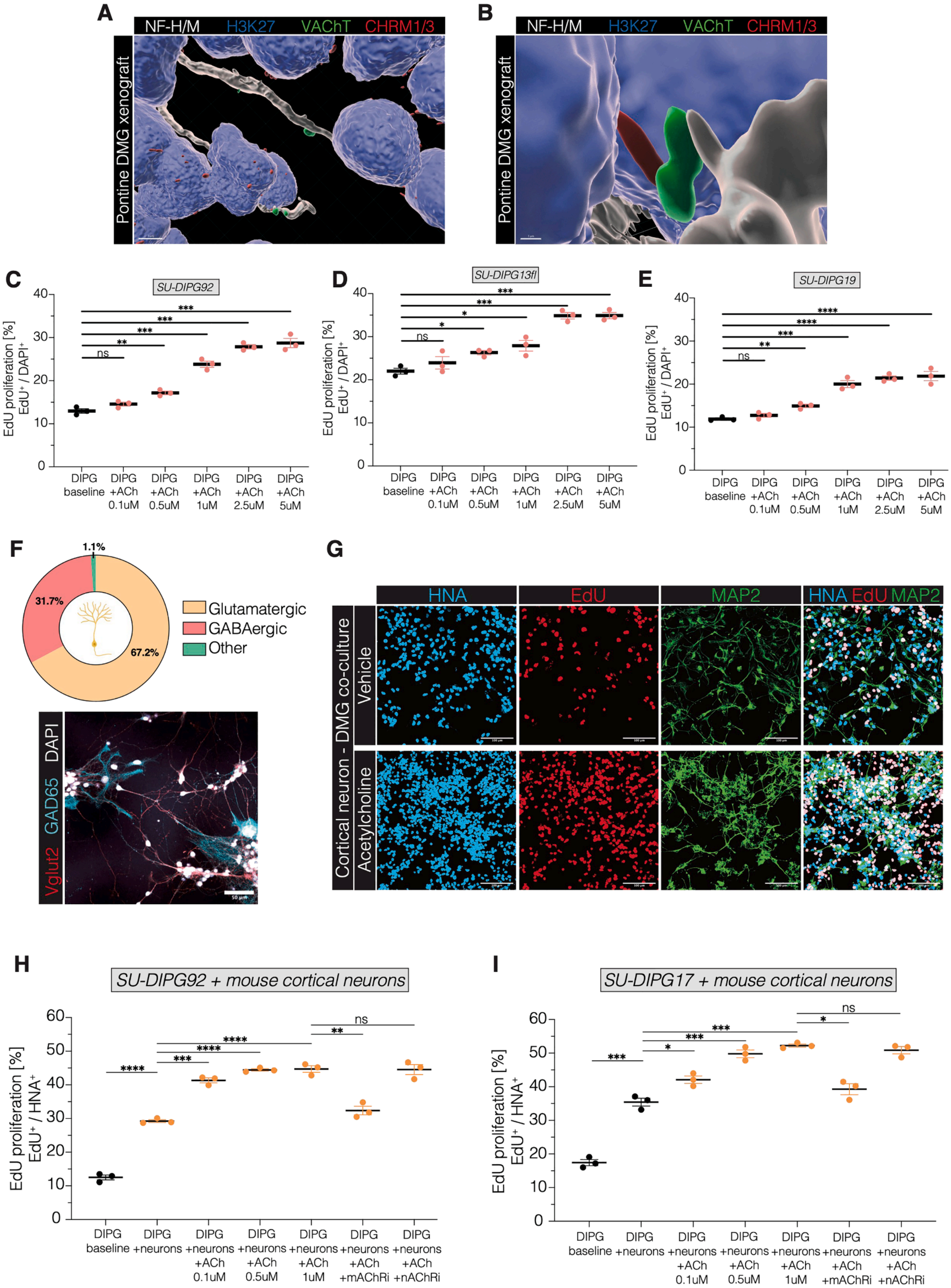

Figure S7. Acetylcholine promotes DMG cell proliferation in monoculture and cortical neuron co-culture in a dose-dependent manner, related to Figure 4

(A and B) 3-dimensional rendering illustrating presynaptic cholinergic neuron (NF-H/M: white) with presynaptic puncta (VAChT: green) co-localizing with post-synaptic puncta (CHRM1/3: red) expressed by post-synaptic glioma cell (H3K27M: blue) in a pontine DMG xenograft. Scale bars, 3 μm (left image) and 1 μm (right image).

(legend continued on next page)

(C–E) Proliferation index (EdU+/DAPI+) of three patient-derived H3K27M DMG cell cultures (C: SU-DIPG92, D: SU-DIPG13fl, E: SU-DIPG19) after exposure to different concentrations of acetylcholine. One-way analysis of variance (ANOVA) with Tukey's post hoc analysis; $*p < 0.05$, $**p < 0.01$, $***p < 0.001$, $****p < 0.0001$, ns: non-significant. Data = mean ± SEM; n = three independent experiments, each with three wells per condition; each data point represents the mean of three wells per condition for a given experiment.

(F) Proportions of neuronal subpopulations as well as representative confocal micrograph of the cortical neuron cultures from mouse pups used for the co-culture experiment shown in Figure S7G–S7I. DAPI: white, Vglut2: red, GAD65: turquoise, scale bar, 50 μm.

(G) Confocal micrographs representing the proliferative effect of adding acetylcholine (1 μM) to a mouse neuron-glioma co-culture. HNA: blue, EdU: red, MAP2: green, scale bars = 100 μm.

(H and I) Proliferation index (EdU+/HNA+) of patient-derived DMG cell cultures when co-cultured with cortical neurons. One-way ANOVA with Tukey's post hoc analysis; $*p < 0.05$, $**p < 0.01$, $***p < 0.001$, $****p < 0.0001$.

Data = mean ± SEM; n = three independent experiments, each with three wells per condition; each data point represents the mean of three wells per condition for a given experiment.

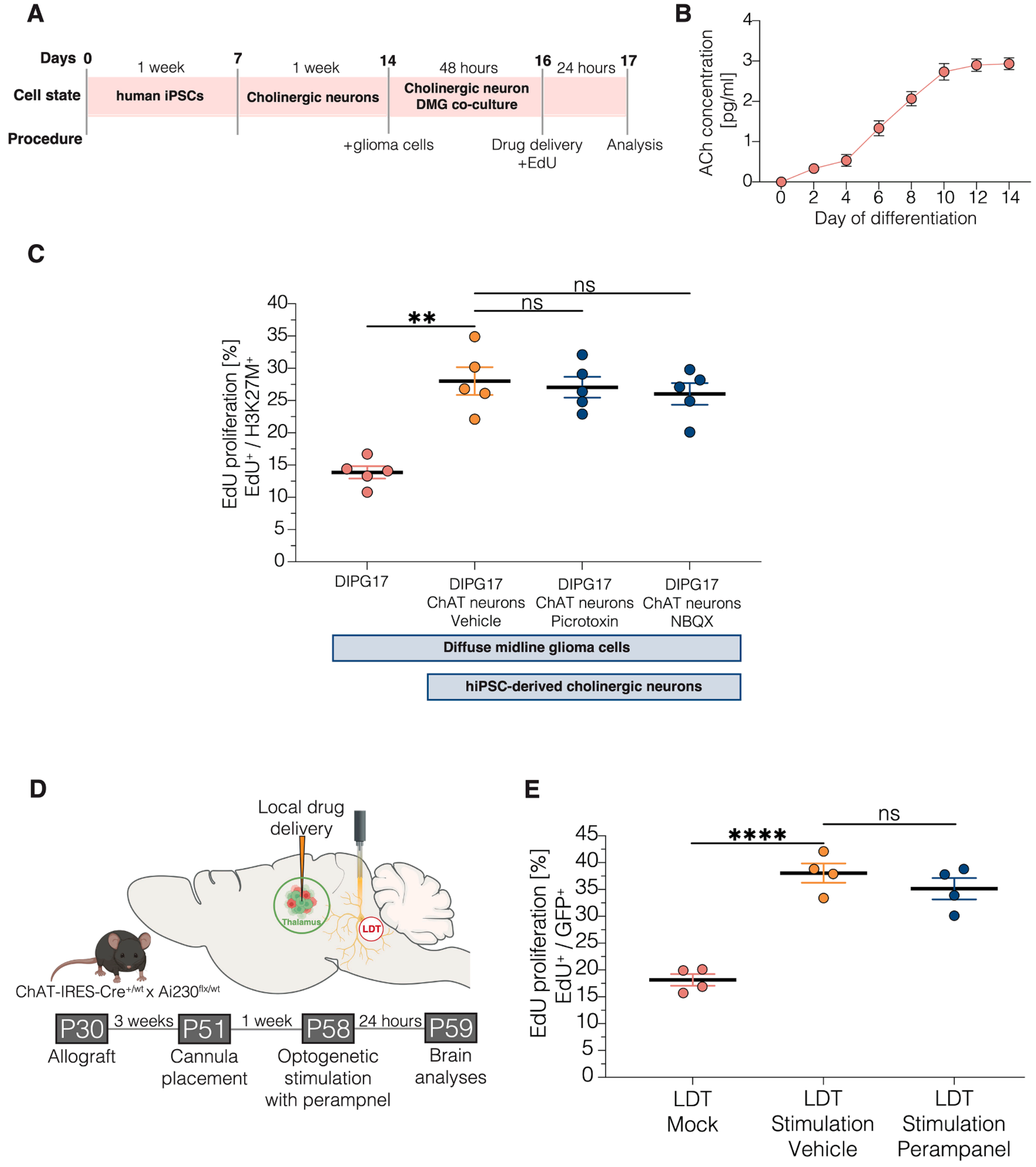

Figure S8. Cholinergic neuronal activity effects on DMG cells are independent of glutamatergic and GABAergic signaling, related to Figure 4

(A) Timeline for the generation of hiPSC-derived cholinergic motor neurons.

(B) Measurement of acetylcholine levels in culture media from hiPSC-derived cholinergic motor neurons over 1 week of differentiation. Data = mean; *n* = three independent experiments, each with three wells per condition; each data point represents the mean of three wells per day of differentiation.

(C) Quantification of the proliferation index (EdU+/H3K27M+) in DMG cells within hiPSC-derived cholinergic neuron-glioma co-cultures, following exposure to picrotoxin (a GABAA receptor antagonist), NBQX (an AMPA receptor antagonist), or vehicle control. One-way analysis of variance (ANOVA) with Tukey's post hoc analysis; $^{**}p < 0.01$, ns = non-significant. Data = mean ± SEM; *n* = five independent experiments, each with three wells per condition; each data point represents the mean of three wells per condition for a given experiment.

(D) Schematic of the experimental paradigm for local delivery of perampanel (AMPA receptor antagonist) to thalamic allografted DMG cells, with simultaneous optogenetic stimulation of the LDT.

(E) Proliferation index (EdU+/GFP+) in thalamic DMG cells, following local delivery of perampanel or vehicle control with simultaneous LDT stimulation (*n* = 4 mice/group). One-way ANOVA with Tukey's post hoc analysis; $^{****}p < 0.0001$, ns = non-significant. Data = mean ± SEM.

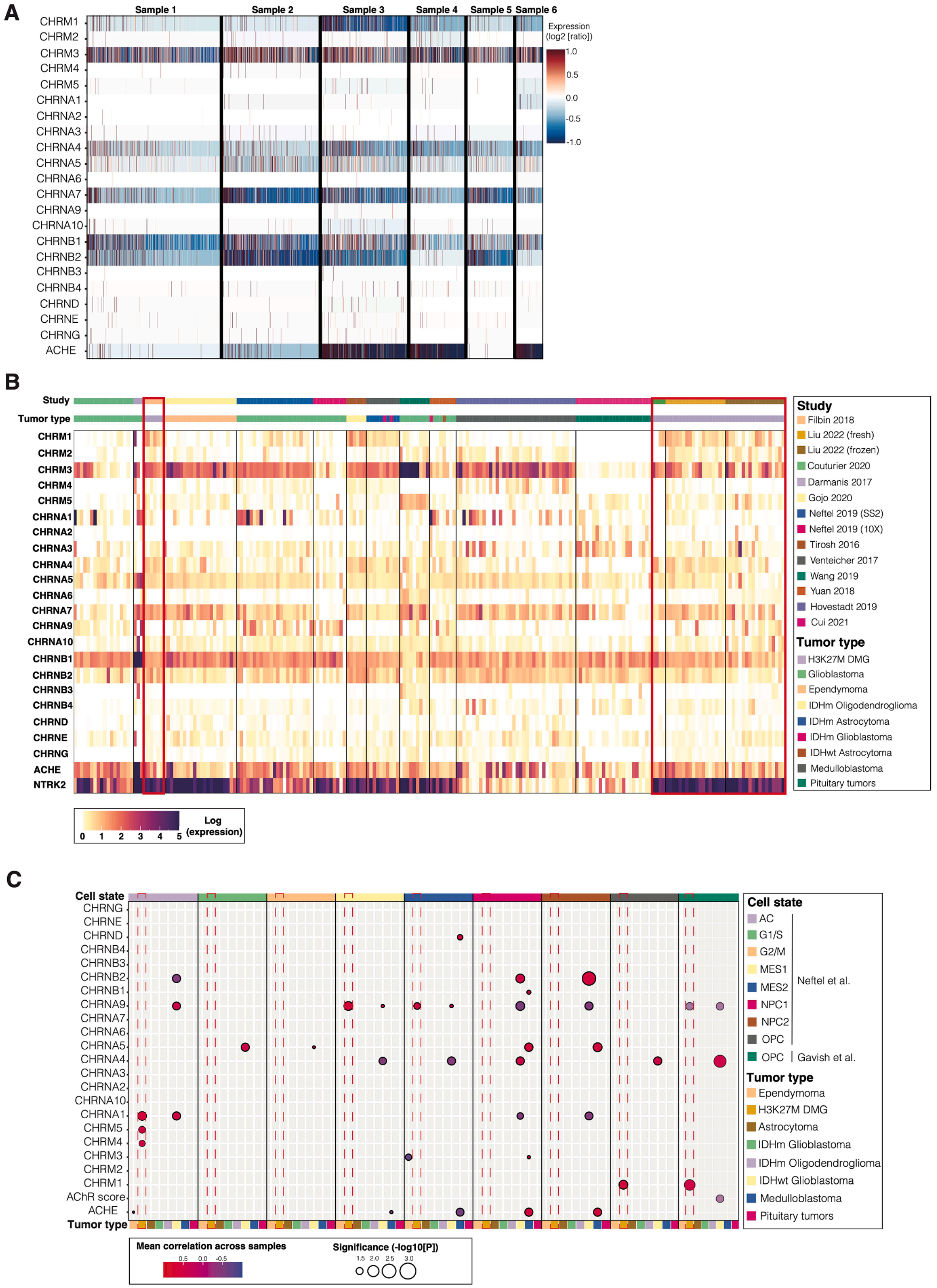

Figure S9. Muscarinic targets for DMG therapy, related to Figure 5

(A) Heatmap illustrating intra- and intertumoral heterogeneity of acetylcholine receptor gene expression in malignant cells from six scRNA-seq DMG samples (Filbin dataset15). The data were centered across all cells per sample.

(legend continued on next page)

(B) Heatmap of pseudo-bulk analysis of cholinergic receptor gene expression in malignant cells across central nervous system tumors from various studies with single-cell or single-nucleus RNA sequencing data. The red-marked boxes indicate studies with DMG samples.

(C) Correlation of cholinergic receptor gene expression with different malignant cell like-states of malig (AC, G1/S, G2/M, MES-1, MES-2, NPC-1, NPC-2, and OPC) in all available CNS tumor entities. The radius of each circle corresponds to the $-\log10(p$ value), and the circle filling is the correlation. Non-significant p values are not shown. Red dashed boxes indicate studies containing DMG samples.

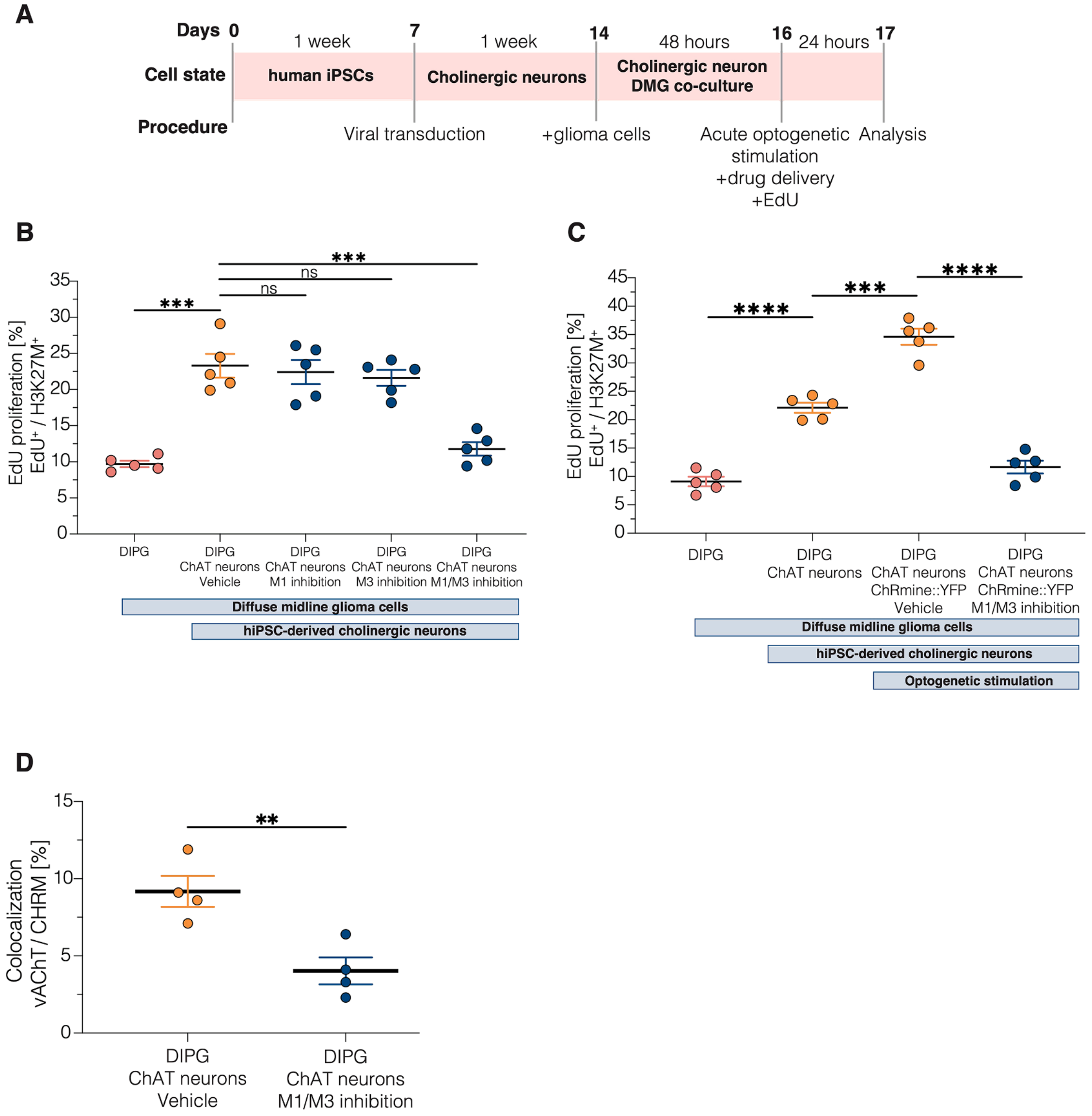

Figure S10. Pharmacological inhibition of CHRM1 and CHRM3 abolishes the effects of cholinergic neuronal activity on DMG cells *in vitro*, related to Figure 5

(A) Timeline for the generation of hiPSC-derived cholinergic motor neurons and co-culturing with glioma cells.

(B and C) Proliferation index (EdU+/H3K27M+) of a patient-derived DMG cell line (SU-DIPG17) when co-cultured and treated with M1 (VU0255035) and M3 (4-DAMP) receptor antagonists, or vehicle control. One-way analysis of variance (ANOVA) with Tukey's post hoc analysis; ****$p < 0.0001$, ***$p < 0.001$, ns = non-significant. Data = mean ± SEM; n = five independent experiments, each with three wells per condition; each data point represents the mean of three wells per condition for a given experiment.

(D) Co-localization of VAChT (presynaptic cholinergic neurons, indicated by NF-H/M+) and CHRM1/3 (post-synaptic glioma cells, indicated by H3K27M+) in the DMG cell line when co-cultured with hiPSC-derived cholinergic motor neurons and treated with M1 (VU0255035) and M3 (4-DAMP) receptor antagonists, or vehicle control. Unpaired two-tailed Welch's t test; **$p < 0.01$. Data = mean ± SEM; n = four independent experiments, each with three wells per condition; each data point represents the mean of three wells per condition for a given experiment.

CellPress
OPEN ACCESS

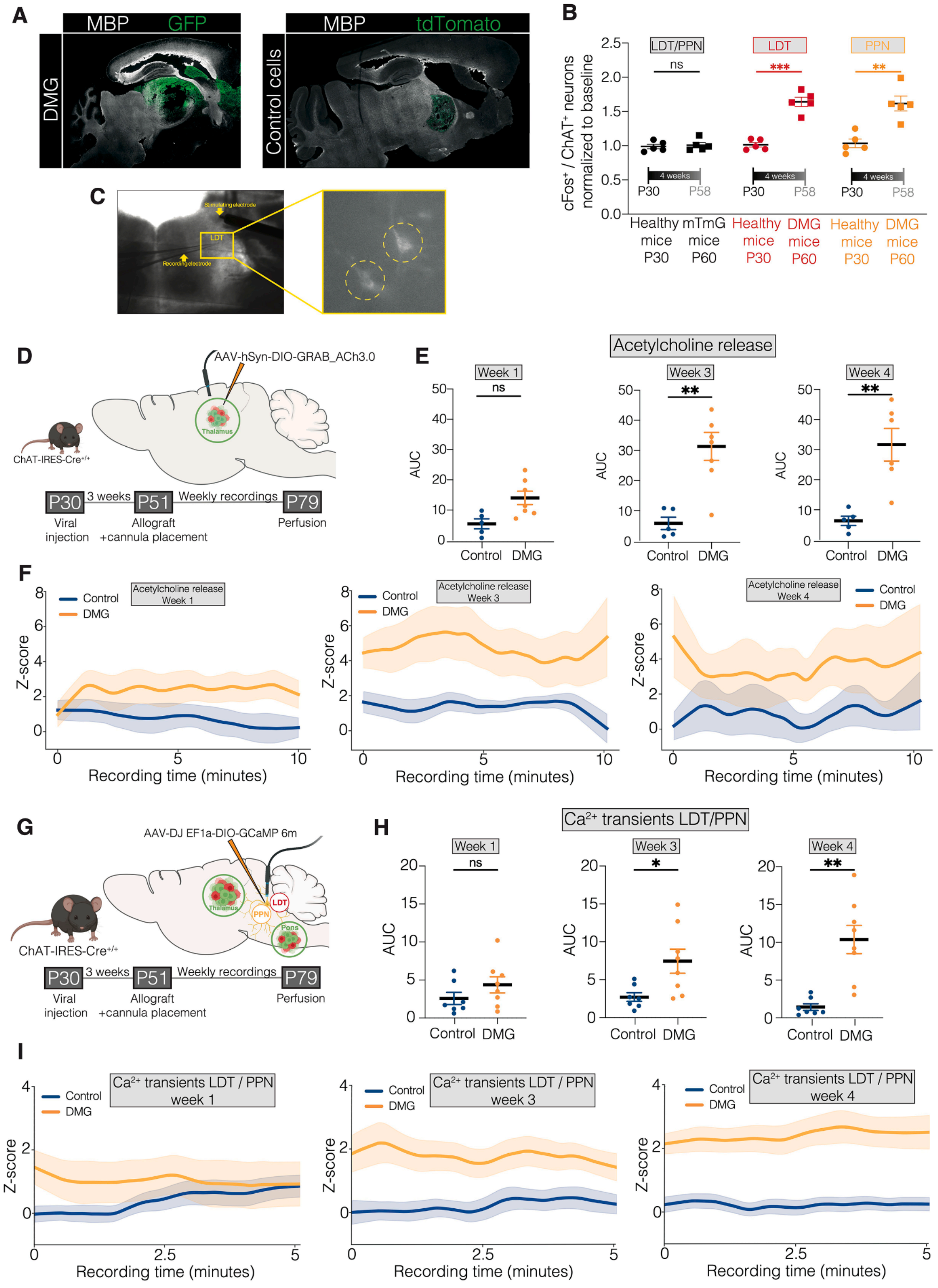

Figure S11. Midbrain cholinergic neurons exhibit increased activity in response to pontine and thalamic DMG cells, related to Figure 6

(A) Confocal micrographs of sagittal sections showing allografted DMG cells (left) and control mTmG cells (right). MBP: white, GFP: green (left image), tdTomato: green (right image).

(legend continued on next page)

(B) Cholinergic neuronal activity in LDT and PPN of thalamic tumor-bearing mice and control-injected mice ($n = 5$ mice/group). Unpaired two-tailed Welch's t test; *$p < 0.05$, **$p < 0.01$, ***$p < 0.001$, ns: non-significant. Data = mean ± SEM.
(C) Bright field image of a brain slice (300 μm thickness) illustrates the LDT region (indicated by the circle). The stimulating electrode was positioned approximately 150 μm above the recording electrode (1 MΩ), which was filled with 1 M NaCl solution. The recording electrode was strategically placed near the densely packed LDT region, which predominantly contains oScarlet-positive cholinergic neurons (right image). A series of calibrated current pulses were administered using an isolated stimulator, with a stimulus duration of 0.1 ms and an amplitude of 10 μA per step.
(D) Schematic of the experimental paradigm for measuring acetylcholine release by cholinergic neurons in the DMG microenvironment.
(E) Area under the curve of GRAB-ACh3.0 recordings from (F) of DMG or control-injected animals. Unpaired two-tailed Welch's t test; **$p < 0.01$, ns = non-significant. Data = mean ± SEM.
(F) Average GRAB-ACh3.0 fluorescence in the peritumoral environment at the 1, 3, and 4-week time point following thalamic implantation of DMG (orange line, $n = 7$ mice at week 1; $n = 6$ mice at week 3 and 4) or control cells (blue line, $n = 5$ mice).
(G) Schematic of the experimental paradigm for measuring calcium transients in cholinergic neurons in LDT and PPN. 4-week-old ChAT-IRES-Cre+/+ mice (P28-30) were injected with AAV-DJ-Ef1a-DIO-GCaMP6m into LDT or PPN and allografted with H3K27M DMG cells or control mTmG cells ("Control") 3 weeks after viral expression. A cannula was placed into LDT or PPN, and mice were recorded weekly.
(H) Area under the curve of fiber photometry recordings of cholinergic neurons from (I) in DMG or control-injected animals. Measured fluorescence is combined from both the LDT and PPN. Unpaired two-tailed Welch's t test; **$p < 0.01$, *$p < 0.05$, ns = non-significant. Data = mean ± SEM.
(I) Fiber photometry recordings showing averaged GCaMP-labeled calcium transients in cholinergic neurons of LDT and PPN from DMG ($n = 8$ mice) or control-injected ($n = 7$ mice) animals at the 1, 3, and 4-week time. Measured fluorescence is combined from both the LDT and PPN.
